# Proteomic identification of prognostic tumour biomarkers, using chemotherapy-induced cancer-associated fibroblasts

**DOI:** 10.18632/aging.100808

**Published:** 2015-10-23

**Authors:** Maria Peiris-Pagès, Duncan L. Smith, Balázs Győrffy, Federica Sotgia, Michael P. Lisanti

**Affiliations:** ^1^ The Breast Cancer Now Research Unit, Institute of Cancer Sciences, University of Manchester, UK; ^2^ The Manchester Centre for Cellular Metabolism (MCCM), Institute of Cancer Sciences, University of Manchester, UK; ^3^ The Cancer Research UK Manchester Institute, University of Manchester, UK; ^4^ MTA TTK Lendület Cancer Biomarker Research Group, Budapest, Hungary; ^5^ Semmelweis University 2nd Dept. of Pediatrics, Budapest, Hungary

**Keywords:** chemotherapy, metabolism, catabolism, cancer-associated fibroblasts, second primary tumours, tumour microenvironment, quantitative proteomics, markers, cancer survival

## Abstract

Cancer cells grow in highly complex stromal microenvironments, which through metabolic remodelling, catabolism, autophagy and inflammation nurture them and are able to facilitate metastasis and resistance to therapy. However, these changes in the metabolic profile of stromal cancer-associated fibroblasts and their impact on cancer initiation, progression and metastasis are not well-known. This is the first study to provide a comprehensive proteomic portrait of the azathioprine and taxol-induced catabolic state on human stromal fibroblasts, which comprises changes in the expression of metabolic enzymes, myofibroblastic differentiation markers, antioxidants, proteins involved in autophagy, senescence, vesicle trafficking and protein degradation, and inducers of inflammation. Interestingly, many of these features are major contributors to the aging process. A catabolic stroma signature, generated with proteins found differentially up-regulated in taxol-treated fibroblasts, strikingly correlates with recurrence, metastasis and poor patient survival in several solid malignancies. We therefore suggest the inhibition of the catabolic state in healthy cells as a novel approach to improve current chemotherapy efficacies and possibly avoid future carcinogenic processes.

## INTRODUCTION

Unlike normal healthy fibroblasts, aged or senescent fibroblasts are pro-tumorigenic [1]. Cellular damage, which is widely considered to be the general cause of aging, occasionally may provide cells with abnormal advantages that can eventually give rise to cancer. Thus, cancer and aging are two different faces of the same underlying process: the accumulation of cellular damage in cells and tissues over the years, which eventually become senescent. Indeed, accumulation of senescent cells has been detected after examination of aged tissues, and it contributes to tissue degeneration during aging [1]. Whether senescence of the stroma is sufficient to initiate tumorigenesis still remains unclear. However, senescent cells can have profound effects on the surrounding microenvironment, through the expression and secretion of a range of pro-inflammatory factors, which is known as the senescence-associated secretory phenotype (SASP). The onset of SASP may help explain the increased tumor incidence observed in aged individuals [2].

The tumour stroma comprises the majority of the neoplastic mass and is mainly composed of fibroblasts [3]. Nevertheless, our comprehension of the tumour microenvironment is rather limited in comparison with that of cancer cells. The emergence of a reactive microenvironment via metabolic stress and inflammation fuels cancer cells, enables tumour growth and invasion, and leads to treatment failure [3–10]. However, the mechanisms by which the metabolic remodelling of cancer-associated stromal fibroblasts (CAFs) regulates the evolution of malignancy or even may control the susceptibility of incompletely transformed cells to become fully malignant are not fully understood.

**Figure 1:**
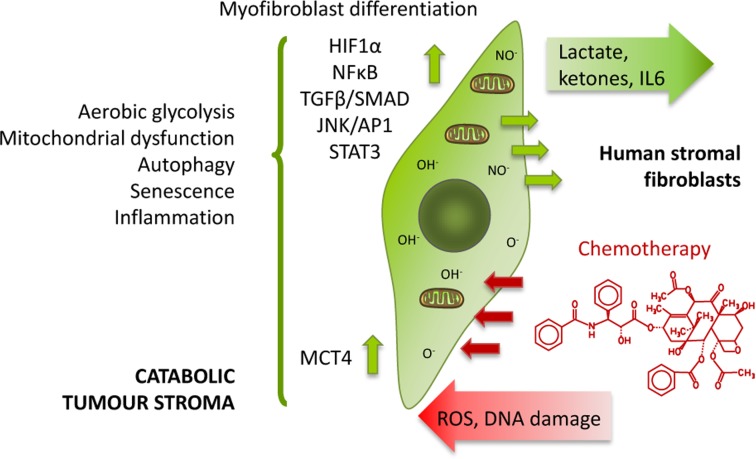
Chemotherapy induces the catabolic tumour stroma phenotype ROS production and DNA damage induced by chemotherapy generate oxidative stress to stromal cells, which in turn brings about several changes in them such as differentiation into CAFs, activation of HIF1α, NFkB, TGFβ, STAT3 or JNK/AP1 signalling pathways, switch to aerobic glycolysis and loss of functional mitochondria, acquisition of an autophagic and senescent phenotype, and release of pro-inflammatory cytokines. Thus, these stromal fibroblasts acquire the catabolic tumour stroma phenotype.

We have previously established that exposure to anticancer agents independently drives metabolic stress and catabolism, autophagy, senescence, myofibroblastic differentiation and production of the pro-inflammatory cytokine Interleukin 6 (IL6) in human stromal fibroblasts *in vitro* (Figure 1) [11]. Thus, according to our model, chemotherapy promotes the same effects in stromal fibroblasts as their interaction with cancer cells, the so-called catabolic tumour stroma phenotype, which creates an energy-rich, pro-inflammatory niche ideal for cancer development and possibly initiation.

Despite the significant number of markers and secreted proteins already related to CAFs, there is little evidence of the contribution of chemotherapy-induced CAF transformation to metastasis or the growth of a second primary tumour after therapy. Indeed, only one report links secretion of factors associated with inflammation and cancer progression in therapy-damaged senescent fibroblasts with *de novo* tumorigenesis [12]. Thus, novel biomarkers are required to improve the prediction of recurrence, metastasis and, in particular, the prediction of therapy-related carcinogenesis. So far, there is one study investigating transcriptomic changes in stromal fibroblasts upon chemotherapeutic treatment, but none investigating phenotypic changes by proteomics [11]. Azathioprine and taxol (paclitaxel) are drugs widely used in chemotherapy for a variety of cancers and in particular taxol is used as the first-line chemotherapeutic agent for ovarian cancer [13–15]. In this study, we describe a strategy based on a label-free quantitative proteomic profiling of fibroblasts obtained after treatment with azathioprine or taxol, which allows us to measure numerous markers of the CAF phenotype. Likewise, the data presented here attempt to identify novel biomarkers of the catabolic remodelling in human stromal fibroblasts that are associated with chemoresistance, metastasis and second primary tumours by reporting their impact on cancer survival. The expression of several over-expressed proteins found in taxol-treated fibroblasts that are involved in metabolism, antioxidant response, autophagy, vesicle trafficking, protein degradation and myofibroblastic transformation correlate with poor prognosis in chemotherapy-treated breast, lung, gastric and ovarian cancer patients. We conclude that a strategy that targets constituents of the tumour microenvironment in combination with conventional chemotherapy may help improving treatment efficacy and avoiding the growth of future malignancies.

## RESULTS

**Figure 2:**
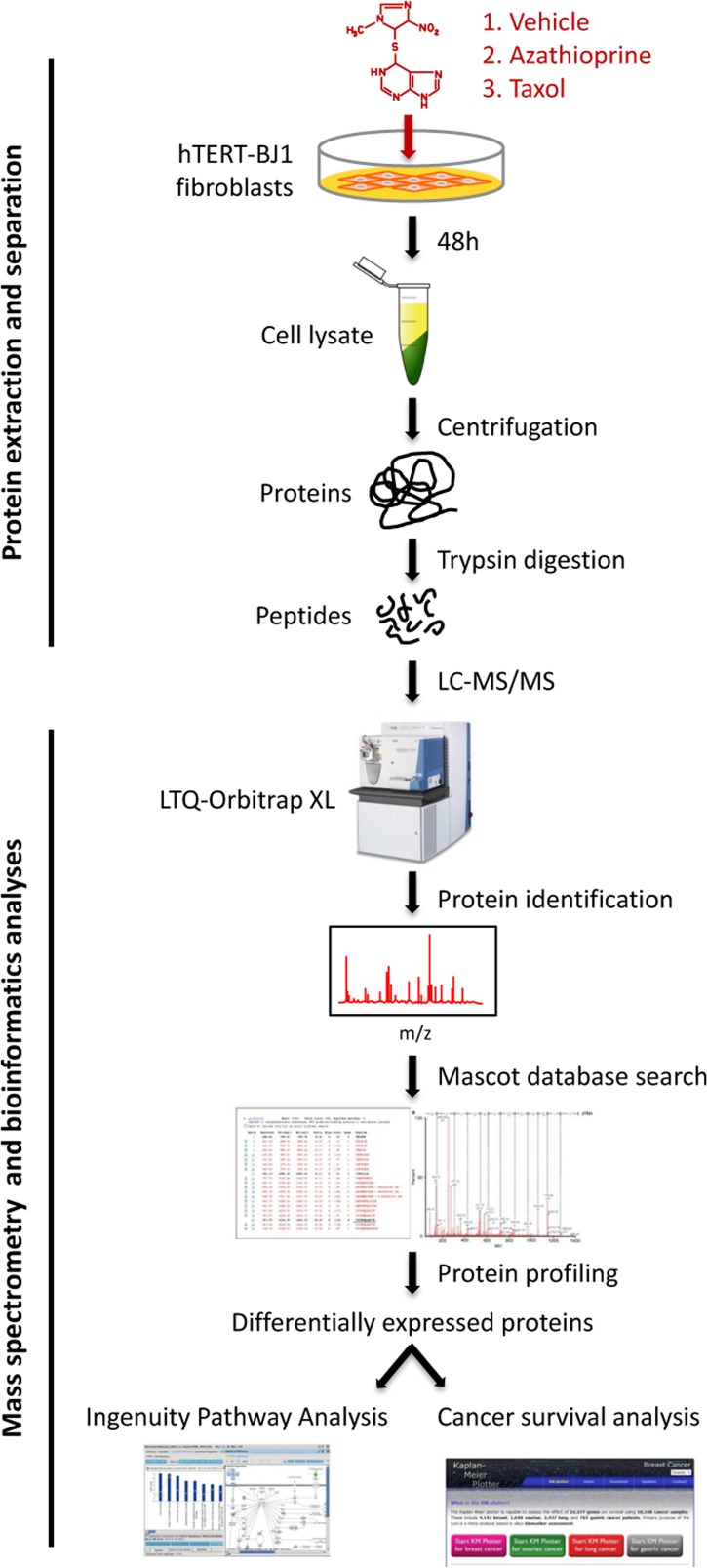
Workflow for the comparative proteome analysis of hTERT-BJ1 fibroblasts treated with azathioprine, taxol or vehicle Protein lysates were obtained from hTERT-BJ1 fibroblasts after 48 h treatment with azathioprine, taxol or vehicle. Peptides obtained after trypsin digestion were analysed via LC-MS/MS on an LTQ-Orbitrap XL mass spectrometer. Label-free quantitative proteomics was used to detect changes in protein abundances across vehicle-treated and drug-treated fibroblast extracts. The proteomics data sets were further analysed using Ingenuity Pathway Analysis and a cancer survival analysis tool (kmplot.com).

**Figure 3:**
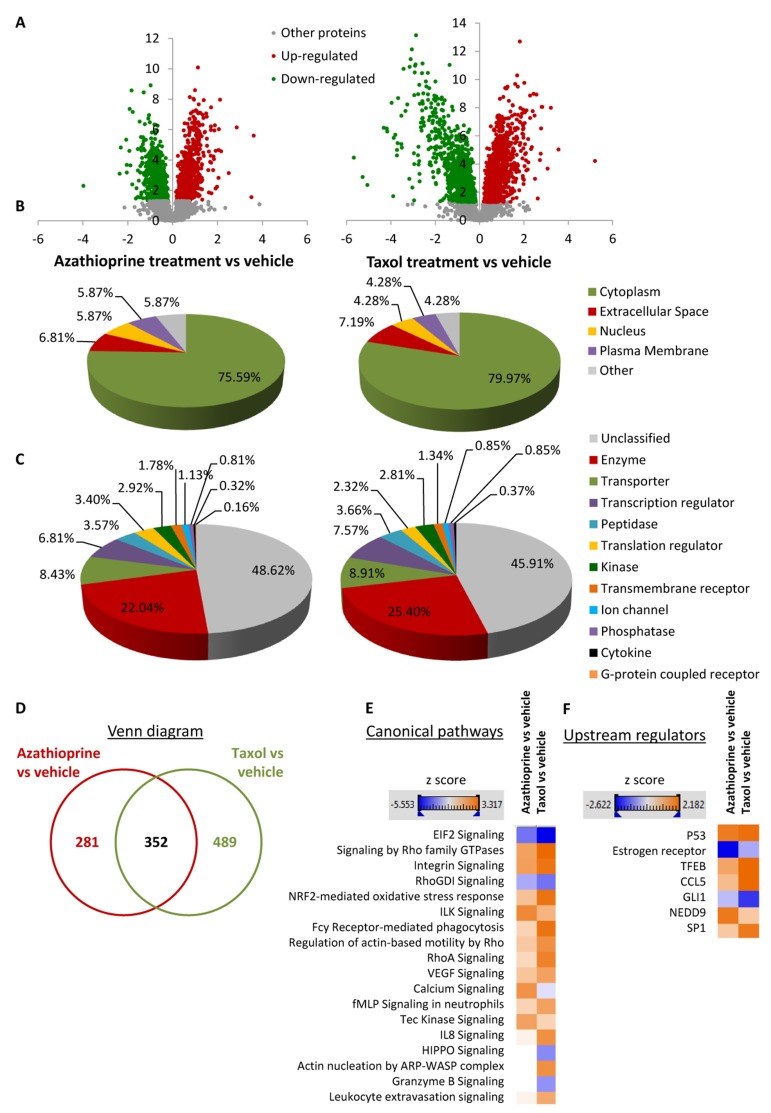
Overview of proteins and pathways identified as differentially regulated in the lysates of azathioprinetreated and taxol-treated fibroblasts relative to vehicle by Ingenuity Pathway Analysis (**A**) Volcano plot representation of protein abundance changes in hTERT-BJ1 fibroblasts upon treatment with azathioprine and taxol compared to vehicle treatment. A total of 1640 differentially regulated proteins with fold changes ≥ 1.15 and *p* values < 0.05 were identified in azathioprine-treated fibroblasts, and 2967 differentially regulated proteins in taxol-treated fibroblasts. X axis represents log2(fold change). Y axis represents −log(p value). Non-significantly regulated proteins are shown in grey, in green, significantly down-regulated proteins and in red, significantly up-regulated proteins. (**B**) Subcellular localization of differentially regulated proteins identified in azathioprine and taxol treatments compared to vehicle treatment. (**C**) Classification of differentially regulated proteins identified in azathioprine and taxol treatments by type. Cellular enzymes account for 22.04% and 25.40% of total differentially regulated proteins identified in azathioprine treatment and taxol treatment, respectively. (**D**) Overlap of differentially regulated proteins identified in azathioprine and taxol treatments compared to vehicle treatment. Of all proteins identified by quantitative proteomics, 352 were proteins the expression of which was found altered in both treatments compared to vehicle. (**E**) Canonical pathways and (**F**) upstream regulators identified or predicted as altered in both treatment conditions compared to vehicle. A positive z score is indicated in orange and points towards an activation of the pathway, and a negative z score, in blue, indicates an inhibition of the pathway.

To identify differentially regulated proteins upon chemotherapeutic treatment, hTERT-BJ1 fibroblasts were exposed for 48 h to either vehicle or sub-lethal concentrations of azathioprine (100 μM) or taxol (100 nM) (Figure S1), and cell lysates were subject to labelfree quantitative proteomics. Following protein digestion with trypsin, peptide fractions were processed on an LTQ-Orbitrap XL mass spectrometer. The experimental workflow used for the present study is depicted in Figure 2. Those peptides identified were further analyzed to find proteomic changes between chemotherapy-treated and vehicle-treated fibroblasts. To define differential regulation, those identified proteins that showed a fold change difference of 1.15 or higher, and *p* values of < 0.05 (ANOVA) compared to vehicle were considered. In the azathioprine-treated fibroblasts, 1640 proteins were identified as differentially expressed, from which 779 were upregulated and 861, down-regulated. In the taxol treatments, 2967 proteins were found as differentially expressed compared to vehicle, from which 1624 were up-regulated and 1343, down-regulated (Figure 3A).

To obtain additional functional insights into pathways that are differentially regulated in stromal fibroblasts upon treatment, bioinformatics analyses of our proteomics datasets were conducted. All differentially expressed proteins were analysed using Ingenuity Pathway Analysis (IPA) to seek altered canonical pathways and toxicity functions. IPA was able to analyse 633 proteins out of 1640 in the azathioprine-treated fibroblasts, and 841 out of 2967 proteins in the taxol-treated fibroblasts. We further examined the subcellular distribution and the nature of these differentially regulated proteins in azathioprine and taxol-treated fibroblasts. Over 80% of all proteins analyzed were intracellular, in particular cytoplasmic proteins (Figure 3B). Likewise, the largest portion of classified proteins, accounting for one fourth of all analysed proteins, were enzymes, over 8% of all proteins were transporter proteins, and over 7% were transcription regulators (Figure 3C). Finally, a comparison analysis revealed that 352 proteins were differentially regulated in both treatment conditions compared to vehicle (Figure 3D).

### Cellular pathways affected by chemotherapy in stromal fibroblasts

#### 1 Metabolism

One of the major contributors to the aging process is mitochondrial dysfunction, which involves a decrease in the oxidative phosphorylation efficacy and an increase in the electron leakage resulting in reduced ATP generation [17]. Similarly, one of the hallmarks of the catabolic tumour stroma is the induction of metabolic stress that favours glycolysis to the detriment of mitochondrial metabolism. We have showed in a previous study that several chemotherapeutic agents, including azathioprine and taxol were able to stimulate stromal fibroblasts to consume more glucose and produce more lactate, which was released via enhanced MCT4 expression, hence increasing extracellular acidification (Figure 1). The cellular ATP content was also minor upon treatment suggesting a decrease in mitochondrial respiration [11].

To validate that chemotherapy-induced glycolytic phenotype and to identify other cellular metabolic pathways potentially altered by chemotherapy, we searched the proteomic data for changes in the expression of metabolic enzymes. Of all differentially regulated proteins, 22.04% and 25.40% were actually enzymes in azathioprine-treated and taxol-treated fibroblasts, respectively (Figure 3C).

The expression of most glycolytic enzymes was significantly altered (Table 1). Interestingly, most of the glycolytic enzymes that were found to be up-regulated are enzymes that perform an irreversible reaction in the glycolytic pathway and all down-regulated enzymes are reversible enzymes, able to perform the opposite reaction, hence also involved in gluconeogenesis. Curiously, LDHA, the enzyme involved in transforming pyruvate into lactate was found to be down-regulated in both treatments suggesting that pyruvate was further processed into the TCA cycle instead of being transformed into lactate.

**Table 1:** Changes in the expression of enzymes involved in glucose metabolism after treatment with azathioprine and taxol for 48 h as measured by quantitative proteomics

**GLYCOLYSIS**		**Azathioprine**	**Taxol**
Solute carrier family 2 (facilitated glucose transporter), member 1 (GLUT1)	SLC2A1	↑ 1.18	
Hexokinase	HK1		↑ 1.43
	HK2	↑ 2.46	
Glucose-6-phosphate isomerase	GPI		↓ 1.27
6-phosphofructokinase type C	PFKP		↑ 1.56
Fructose-bisphosphate aldolase A	ALDOA	↑ 2.10	↓ 11.58
Glyceraldehyde-3-phosphate dehydrogenase	GAPDH	↑ 1.90	↑ 1.89
Triosephosphate isomerase 1	TPI1		↑ 1.33
Phosphoglycerate kinase 1	PGK1		↓ 1.58
Phosphoglycerate mutase	PGAM1		↓ 1.38
	PGAM4	↓ 1.45	
Enolase 1	ENO1	↓ 1.37	
Pyruvate kinase	PKM		↑ 1.65
**POST-GLYCOLYSIS PROCESSES**		**Azathioprine**	**Taxol**
L-lactate dehydrogenase A chain	LDHA	↓ 1.47	↓ 1.46
Monocarboxylate transporter 4 (MCT4)	SLC16A3		↑ 2.21
Pyruvate dehydrogenase	PDHB	↓ 1.20	↓ 1.40
**GLUCONEOGENESIS**		**Azathioprine**	**Taxol**
Glucose-6-phosphate isomerase	GPI		↓ 1.27
Aldolase A, fructose-bisphosphate	ALDOA	↑ 2.10	↓ 11.57
Glyceraldehyde-3-phosphate dehydrogenase	GAPDH	↑ 1.90	↑ 1.89
Phosphoglycerate kinase 1	PGK1		↓ 1.58
Phosphoglycerate mutase	PGAM1		↓ 1.38
	PGAM4	↓ 1.45	
Enolase 1	ENO1	↓ 1.37	
Malate dehydrogenase	MDH1		↓ 1.80
	MDH2		↓ 1.23
Malic enzyme 2, NAD(+)-dependent, mitochondrial	ME2		↓ 1.62
**PENTOSE PHOSPHATE PATHWAY**		**Azathioprine**	**Taxol**
**Glucose-6-phosphate dehydrogenase**	**G6PD**	↓ 2.03	↑ 2.69
	H6PD	↑ 1.46	
6-phosphogluconolactonase	PGLS		↑ 1.54
Phosphogluconate dehydrogenase	PGD		↓ 1.57
Transketolase	TKT	↓ 1.88	
Transaldolase	TALDO	↑ 2.13	
**HEXOSAMINE BIOSYNTHESIS PATHWAY**		**Azathioprine**	**Taxol**
Glutamine-fructose-6-phosphate transaminase 1	GFPT1		↑ 1.36
**Glucosamine-6-phosphate deaminase 1**	**GNPDA1**		↑ 2.46

Chemotherapy increased the expression of several enzymes involved in glycolysis, pentose phosphate pathway, and hexosamine biosynthesis. Enzymes that show amplified expression after treatment are shown in red, and enzymes that are decreased are shown in green. Number indicates the fold increase or fold decrease in protein expression in chemotherapy-treated versus vehicle-treated hTERT-BJ1 fibroblasts.

**Figure 4:**
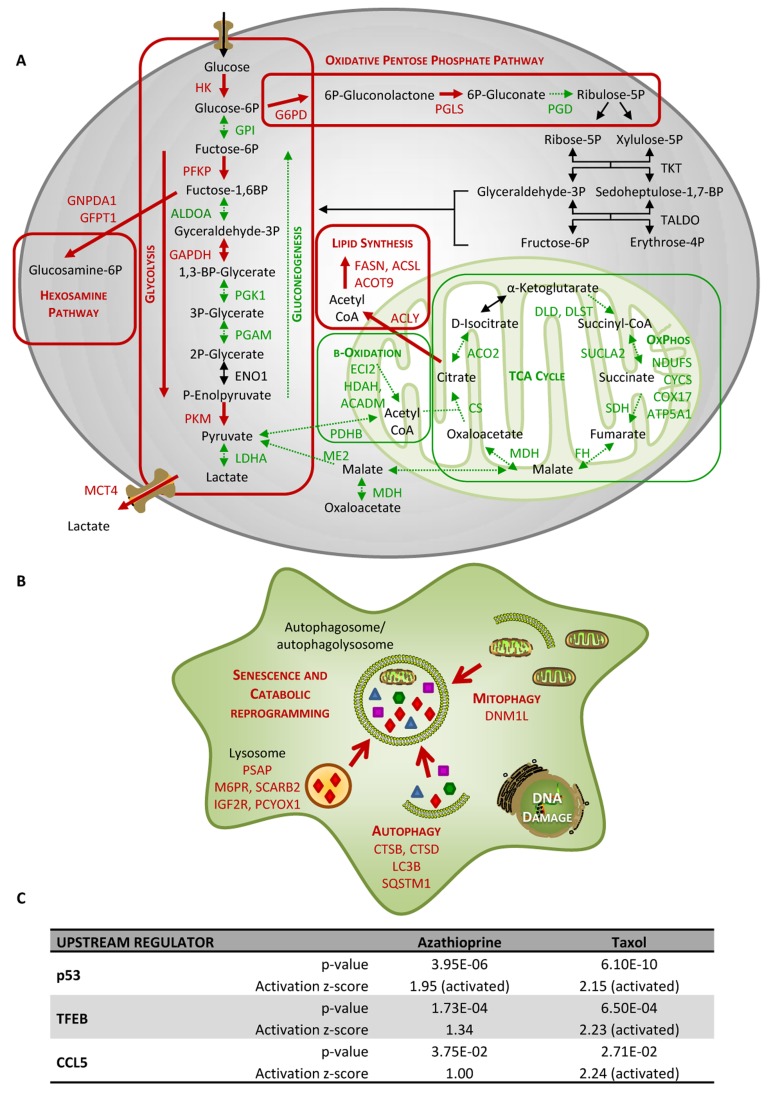
Taxol treatment alters several cellular metabolic pathways and induces autophagy and senescence in hTERTBJ1 fibroblasts (**A**) Summary of changes observed in the expression of numerous metabolic enzymes after treatment with taxol for 48 h, as measured by quantitative proteomics. Taxol treatment increased the expression of enzymes involved in glycolysis, pentose phosphate, and hexosamine biosynthesis and lipid synthesis pathways in detriment of those involved in gluconeogenesis and mitochondrial metabolism (TCA cycle, oxidative phosphorylation, and mitochondrial β–oxidation). Enzymes that show amplified expression after taxol treatment are shown in red, and enzymes that are decreased are shown in green. Cellular metabolic pathways that may be up-regulated are boxed in red, and those that may be down-regulated are boxed in green. (**B**) Summary of changes observed in the expression of numerous autophagy and senescence-related proteins after treatment with taxol for 48 h, as measured by quantitative proteomics. Taxol amplified the expression of proteins involved in senescence, autophagy, mitophagy and vesicle formation and trafficking. Proteins that show increased expression after treatment are shown in red. (**C**) Ingenuity Pathway Analysis of azathioprine and taxol-treated hTERT-BJ1 fibroblasts predicted p53, TFEB and CCL5 to be activated in these cells.

Nevertheless, LDH is an enzyme that exhibits feedback inhibition, by which high lactate concentrations can suppress it. In fact, MCT4, the monocarboxylate transporter responsible for the secretion of lactate, turned out to be up-regulated in taxol-treated hTERT-BJ1 cells, and regarding the further processing of pyruvate into acetyl-CoA and the citric acid (TCA) cycle, the expression of practically all enzymes were found to be down-regulated in taxol-treated fibroblasts compared to vehicle indicating a dramatic down-regulation of mitochondrial metabolism. A similar down-regulation trend was observed in numerous proteins involved in all oxidative phosphorylation complexes (Table 2) and mitochondrial function proteins (Table S1). Finally, several enzymes involved in mitochondrial fatty acid β-oxidation were also identified as down-regulated, and a few enzymes involved in fatty acid biosynthesis, up-regulated (Table S2). Other metabolic changes included enzymes of the oxidative pentose phosphate pathway, responsible for the generation of antioxidant power (NADPH), the hexosamine synthesis pathway, accountable for the production of amino sugars used for the synthesis of glycoproteins, glycolipids and proteoglycans, and a few enzymes involved in the generation of ketone bodies (Table 1 and 5). Figure 4A summarises all changes observed in the expression of metabolic enzymes in taxol-treated fibroblasts and their contribution to different cellular metabolic pathways.

**Table 2:** Changes in the expression of enzymes involved in mitochondrial glucose metabolism after treatment with azathioprine and taxol for 48 h as measured by quantitative proteomics

**TCA CYCLE**		**Azathioprine**	**Taxol**
Citrate synthase, mitochondrial	CS		↓ 1.52
Aconitate hydratase	ACO2	↑ 1.41	↓ 1.23
Dihydrolipoyl dehydrogenase	DLD		↓ 1.48
Alpha-ketoglutarate dehydrogenase complex dihydrolipoyl succinyltransferase	DLST	↓ 1.23	↓ 1.70
2-oxoglutarate dehydrogenase	OGDH	↓ 1.37	
Beta-succinyl CoA synthetase	SUCLA2		↓ 1.25
Succinate dehydrogenase complex	SDHA		↓ 2.43
	SDHB	↓ 1.51	
Fumarate hydratase	FH		↓1.27
Malate dehydrogenase	MDH1		↓ 1.80
	MDH2		↓ 1.22
**OXIDATIVE PHOSPHORYLATION**		**Azathioprine**	**Taxol**
NADH dehydrogenase (complex I)	NDUFV1		↑ 1.54
	NDUFV2		↓ 1.45
	NDUFS1		↓ 1.73
	NDUFS7		↓ 1.31
	NDUFB10		↓ 1.29
Succinate dehydrogenase complex (complex II)	SDHA		↓ 2.43
	SDHB	↓ 1.51	
Coenzyme Q – cytochrome c reductase (complex III)	CYCS		↓ 1.61
	UQCRC1		↓ 1.42
	UQCRB		↑ 1.62
Cytochrome c oxidase (complex IV)	COX17		↓ 2.00
	COX6C		↓ 1.62
	COX6A1	↑ 1.47	
	COX5A		↓ 1.39
	COX5B	↑ 1.37	
ATP synthase (complex V)	ATP5A1	↓ 1.51	↓ 1.16
	ATP5F1	↓ 1.70	↓ 1.51
	ATP5H	↑ 1.56	
	ATP5J	↑ 1.36	
	ATP5B		↑ 2.36

Taxol treatment remarkably decreased the expression of enzymes of the TCA cycle and oxidative phosphorylation. Enzymes that show increased expression after treatment are shown in red, and enzymes that are decreased are shown in green. Number indicates the fold increase or fold decrease in protein expression in chemotherapy-treated versus vehicle-treated hTERT-BJ1 fibroblasts.

**Figure 5:**
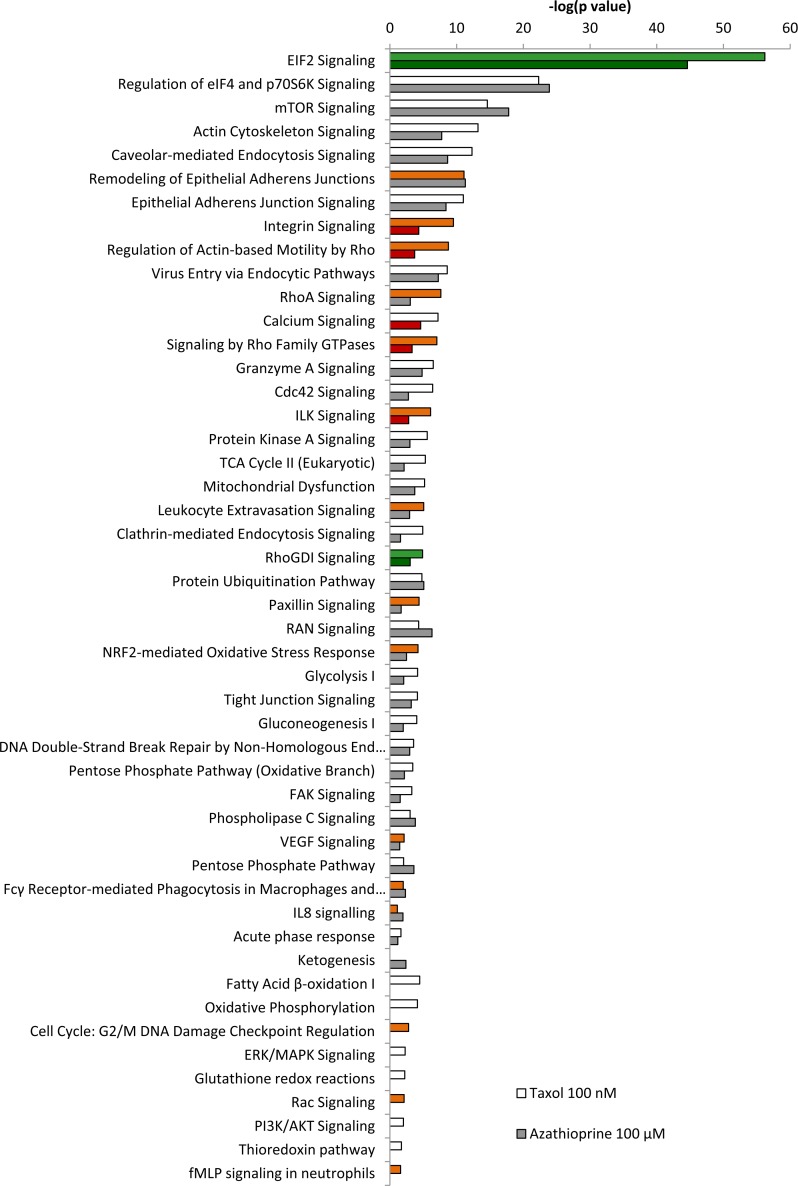
Pathway analysis of differentially expressed proteins in hTERT-BJ1 fibroblasts treated with azathioprine or taxol compared to vehicle-treated cells Ingenuity Pathway Analysis showed canonical pathways significantly altered by the proteins differentially expressed in hTERT-BJ1 fibroblasts treated with azathioprine or taxol (*P* < 0.05). The *p* value for each pathway is indicated by the bar and is expressed as −1 times the log of the *p* value. Green coloured bars show a predicted inhibition of the pathway (z score <-1.9) and red coloured bars indicate a predicted activation of the pathway (z score > 1.9).

**Figure 6:**
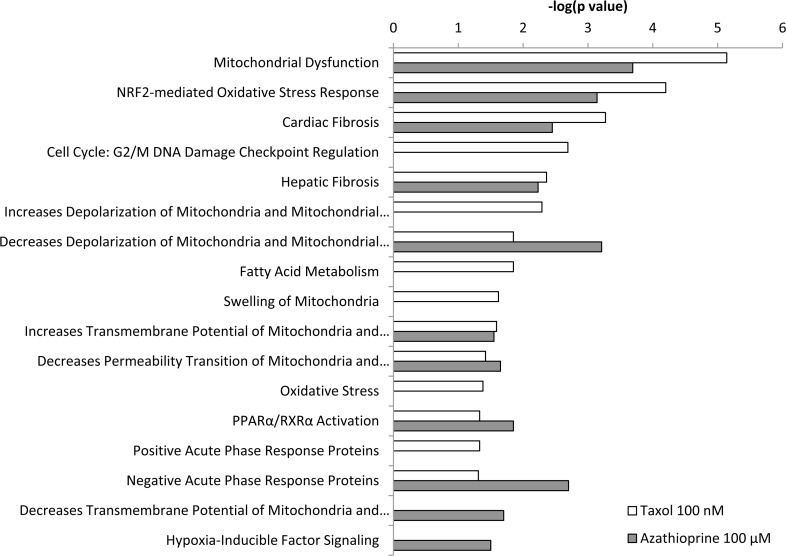
Toxicity effects of differentially expressed proteins in hTERT-BJ1 fibroblasts treated with azathioprine or taxol compared to vehicle-treated cells Ingenuity Pathway Analysis showed toxicity functions significantly enriched by the proteins differentially expressed in hTERT-BJ1 fibroblasts treated with azathioprine or taxol (*P* < 0.05). The *p* value for each pathway is indicated by the bar and is expressed as −1 times the log of the *p* value.

IPA revealed glycolysis, gluconeogenesis and pentose phosphate pathway as altered canonical pathways in both azathioprine and taxol treatments. Similarly, IPA revealed mitochondrial dysfunction, TCA cycle, and PPARα/RXRα activation, responsible for ketone body production and fatty acid metabolism, as two of the top canonical pathways affected by both treatments, and oxidative phosphorylation and fatty acid β-oxidation, as altered pathways also in taxol-treated cells (Figure 5 and Table S5). The toxicity impact of both drugs extensively involved mitochondrial dysfunction and damage, and also fatty acid metabolism and PPARα/RXRα activation (Figure 6 and Table S6).

To recapitulate, the previously identified chemotherapy-induced metabolic stress in stromal fibroblasts is clearly detected by quantitative proteomics and it represents not only an increase in glycolysis and a reduction in mitochondrial function, as observed in our previous study, but also affects other metabolic pathways, including hexosamine synthesis and pentose phosphate pathways, fatty acid metabolism and ketogenesis.

#### 2 Antioxidant response and stress-related pathways

Another feature of the catabolic tumour stroma is the induction of oxidative stress, which can be induced by chemotherapy in cancer cells and also healthy tissues (Figure 1) [18, 19]. Particularly, we previously showed increased ROS production and antioxidant response in hTERT-BJ1 fibroblasts after treatment with taxol [11]. We have seen in this study that several enzymes of the oxidative branch of the pentose phosphate pathway, which is responsible for the generation of antioxidant power (NADPH), were up-regulated in chemotherapy-treated stromal fibroblasts. Thus, we sought for other antioxidant response proteins in the proteomics datasets. The expression of numerous proteins involved in Nrf2-mediated antioxidant response and other antioxidant proteins was significantly altered, in most cases up-regulated (Table 3), suggesting an activation of the pathway in both azathioprine and taxol-treated hTERT-BJ1 fibroblasts, although it was found to be notably higher in taxol-treated cells.

**Table 3:** Changes in the expression of Nrf2-target proteins and other proteins involved in the antioxidant response after treatment with azathioprine and taxol for 48 h as measured by quantitative proteomics and indicated by IPA analysis

**NRF2-MEDIATED ANTIOXIDANT RESPONSE**		**Azathioprine**	**Taxol**
ATP-binding cassette, sub-family C (CFTR/MRP), member 1	ABCC1	↓ 2.34	
Actin, alpha 2, smooth muscle, aorta	ACTA2	↑ 1.34	↑ 1.78
Actin, beta	ACTB		↑ 1.30
Actin, alpha, cardiac muscle 1	ACTC1	↑ 1.75	↑ 1.20
Carbonyl reductase 1	CBR1		↑ 1.29
Chaperonin containing TCP1, subunit 7 (eta)	CCT7	↑ 2.89	
DnaJ (Hsp40) homolog	DNAJA1	↑ 1.61	↓ 9.61
	DNAJB11	↓ 1.34	
	DNAJC8	↓ 1.79	↓ 1.58
	DNAJC13		↓ 4.30
Epoxide hydrolase 1, microsomal (xenobiotic)	EPHX1		↑ 2.06
Ferritin, light polypeptide	FTL	↑ 1.61	↑ 6.97
Glutathione S-transferase	GSTK1		↑ 1.34
	GSTO1		↑ 1.57
	GSTP1		↓ 1.53
Heme oxygenase (decycling) 1	HMOX1	↑ 4.48	↑ 2.83
Mitogen-activated protein kinase 3	MAPK3	↑ 1.54	
Peptidylprolyl isomerase B (cyclophilin B)	PPIB	↑ 1.60	↑ 1.36
Peroxiredoxin 1	PRDX1		↑ 1.68
Protein kinase C, alpha	PRKCA	↑ 1.96	
Related RAS viral (r-ras) oncogene homolog	RRAS		↑ 2.44
Superoxide dismutase 1, soluble	SOD1		↑ 1.48
Superoxide dismutase 2, mitochondrial SOD2		↑ 1.69	↓ 1.87
Sequestosome 1	SQSTM1		↑ 3.50
Thioredoxin TXN			↑ 2.75
Thioredoxin reductase 1	TXNRD1		↑ 2.66
Valosin-containing protein VCP		↓ 1.29	
**ANTIOXIDANTS (OTHER)**		**Azathioprine**	**Taxol**
Aminopeptidase N	ANPEP	↑ 1.78	↑ 1.56
Glutaredoxin-1	GLRX		↑ 1.25
Glutathione peroxidase	GPX8		↓ 1.85
Peroxidasin	PXDN		↑ 3.57
	PRDX4	↑ 1.50	
Peroxiredoxin	PRDX5		↑ 1.33
	PRDX6		↓ 1.23

Chemotherapy mostly increased the expression of proteins involved in antioxidant response, mainly in taxol-treated fibroblasts. Proteins that show amplified expression after treatment are shown in red, and proteins that are decreased are shown in green. Number indicates the fold increase or fold decrease in protein expression in chemotherapy-treated versus vehicle-treated hTERT-BJ1 fibroblasts.

A higher presence of antioxidant response proteins was further confirmed using IPA, which revealed Nrf2-mediated antioxidant response to be one of the top canonical pathways affected by azathioprine and taxol treatments, although z score values demonstrated a significant activation of this pathway exclusively in taxol-treated fibroblasts. Similarly, taxol-treated cells showed altered glutathione and thioredoxin antioxidant pathways (Figure 3E and 5, and Table S5). The toxicity of these chemotherapeutic drugs also involved oxidative stress. HIF signalling, which is activated in response to stress and a central player in the regulation of cellular metabolism, was also identified as a toxic effect of azathioprine treatment (Figure 6 and Table S6), and had been previously identified as activated in hTERT-BJ1 fibroblasts treated with azathioprine and taxol [11]. Thus, proteomics analysis clearly detects an activation of the antioxidant response after treatment with chemotherapy.

#### 3 Myofibroblastic differentiation

Cancer-associated fibroblasts are commonly identified by their expression of alpha smooth muscle actin (αSMA) [20] (Figure 1). Indeed, we previously showed up-regulation of αSMA by immunoblotting in taxol-treated hTERT-BJ1 [11]. Quantitative proteomics profiling of azathioprine and taxol-treated hTERT-BJ1 fibroblasts also revealed a significantly higher presence of αSMA and many other myofibroblastic markers, such as fibroblast activation protein (FAP) or vimentin, as well as muscle-related proteins compared to the vehicle-treated control (Table 4).

**Table 4:** Changes in the expression of proteins involved in myofibroblastic transformation and muscle-related proteins after treatment with azathioprine and taxol for 48 h as measured by quantitative proteomics

CANCER-ASSOCIATED FIBROBLAST AND MUSCLE-RELATED PROTEINS		Azathioprine	Taxol
Actin, alpha, cardiac muscle	ACTC1	↑ 1.75	↑ 1.20
Actin, alpha 2, aortic smooth muscle (αSMA)	ACTA2	↑ 1.34	↑ 1.78
Caldesmon	CALD1		↑ 2.49
Calponin	CNN1		↑ 2.76
	**CNN2**	↑ 1.81	↑ 2.76
	CNN3	↓ 1.28	↑ 2.46
Desmin	DES	↑ 1.21	↓ 1.79
Dysferlin	DYSF		↑ 1.24
Fibroblast activation protein	FAP	↑ 1.74	↑ 1.56
Fibronectin 1	FN1	↑ 2.02	↑ 2.03
Filamin	FLNA	↓ 1.92	↑ Infinity
	FLNB	↓ 1.23	
	FLNC		↑ 1.56
Moesin	MSN	↑ 1.37	↑ 1.60
Myoferlin MYOF		↑ 4.15	↑ 1.95
Myosin	MYH4		↑ 1.62
	MYH9	↑ 1.64	
	MYH10	↑ 1.37	
	MYH11	↑ 1.47	↑ 1.90
	MYH14	↓ 3.06	
	MYO1B		↓ 1.72
	MYO1C	↑ 2.21	↑ 1.59
	MYO10	↑ 1.37	
	MYO18B	↓ 1.50	↑ 2.62
Myosin, light polypeptide kinase	MYLK		↑ 1.88
Myosin phosphatase Rho interacting protein	MPRIP		↑ 1.45
Myosin regulatory light polipeptides	MYL6	↑ 1.33	↑ 1.74
	MYL9		↑ 1.77
	MYL12A		↑ 2.21
Palladin	PALLD		↑ 3.88
Platelet-derived growth factor receptor, beta	PDGFRB		↑ 1.59
Prolyl 4-hydroxylase	P4HA1		↑ 3.14
	P4HA2	↓ 1.32	↓ 2.50
	P4HB	↓ 1.76	
Talin	**TLN1**	↑ 1.46	↑ 2.95
	TLN2	↓ 1.80	
Transgelin 2	TAGLN2	↑ 1.93
Tropomyosin	TPM1	↓ 2.63	↓ 4.23
	TPM2	↑ 1.24	
	TPM3	↑ 1.43	
	TPM4		↑ 2.12
Vimentin	VIM	↑ 1.72	↑ 1.76

Chemotherapy enormously increased the expression of proteins involved in CAF transformation. Proteins that show amplified expression after treatment are shown in red, and proteins that are decreased are shown in green. Number indicates the fold increase or fold decrease in protein expression in chemotherapy-treated versus vehicle-treated hTERT-BJ1 fibroblasts.

Myofibroblasts are mostly responsible for the presence of fibrosis [21], and the toxicity functions of these chemotherapeutic drugs involved tissue fibrosis, as analysed by IPA (Figure 6 and Table S6). Therefore, chemotherapy can independently induce the differentiation of hTERT-BJ1 fibroblasts into cancer-associated fibroblasts, which can be detected by a numerous increase of myofibroblastic markers in quantitative proteomics analysis.

#### 4 Autophagy and senescence

Autophagy and senescence represent a common response to stresses such as exposure to DNA-damaging exogenous cytotoxic agents, including chemotherapy or radiation [22, 23]. Indeed our previous study indicated that azathioprine and taxol induce autophagic vesicle formation and increase β-galactosidase activity in stromal fibroblasts [11]. Thus, to further examine changes in autophagy and senescence upon chemotherapeutic exposure, the differential expression of several autophagy, mitophagy and lysosomal markers was analysed using the proteomics datasets. Numerous autophagy and senescence markers such as sequestrosome 1, also known as p62, cathepsin B or the lysosomal enzyme β-galactosidase were found to be up-regulated in either azathioprine or taxol treatments (Table 5). In addition, DNM1L, a protein involved in mitochondrial fission, was up-regulated in taxol-treated hTERT-BJ1 fibroblasts, and OPA1, a protein involved in mitochondrial fusion, was down-regulated in azathioprine-treated cells. Likewise, VAT1, a vesicle membrane protein that inhibits mitochondrial fusion, was up-regulated in both treatments, suggesting that mitophagy might be also activated in response to chemotherapy (Table 5 and Table S3).

**Table 5:** Changes in the expression of autophagy, mitophagy and senescence markers and ketogenesis enzymes after treatment with azathioprine and taxol for 48 h as measured by quantitative proteomics

**AUTOPHAGY MARKERS**		**Azathioprine**	Taxol
Cathepsin	CATB	↑ 1.56	↑ 1.55
	CATD		↑ 1.62
Lysosomal-associated membrane protein 1	LAMP1	↑ 1.55	
Microtubule-associated protein 1 light chain 3 beta	MAP1LC3B		↑ 1.58
Sequestrosome 1 (p62)	SQSTM1		↑ 3.50
**MITOPHAGY MARKERS**		**Azathioprine**	**Taxol**
Dynamin-1-like protein (fission)	DNM1L		↑ 1.71
Dynamin-like 120 kDa protein, mitochondrial (fusion)	OPA1	↓ 1.44	
**LYSOSOMAL PROTEINS (OTHER)**		**Azathioprine**	**Taxol**
Cation-dependent mannose-6-phosphate receptor	M6PR		↑ 2.38
**Cation-independent mannose-6-phosphate receptor**	**IGF2R**	↑ 3.58	↑ 1.88
Galactosidase, beta 1	GLB1	↑ 1.53	
Late endosomal/lysosomal adaptor, MAPK and mTOR activator 1	LAMTOR1	↑ 1.85	
Lysosome membrane protein 2	SCARB2	↑ 1.63	↑ 1.49
N-acetylglucosamine-6-sulfatase	GNS		↑ 1.43
Prenylcysteine oxidase 1	PCYOX1		↑ 1.55
Prosaposin	PSAP	↑ 2.21	↑ 2.13
**KETOGENESIS**		**Azathioprine**	**Taxol**
Hydroxyacyl-CoA dehydrogenase	HADH		↓ 1.43
	HADHA	↓ 1.57	
	HADHB	↑ 1.36	
3-Hydroxymethyl-3-methylglutaryl-CoA lyase	HMGCL	↑ 1.67	

Chemotherapy incremented the expression of proteins involved in autophagy and senescence. Proteins that show increased expression after treatment are shown in red, and proteins that are decreased are shown in green. Number indicates the fold increase or fold decrease in protein expression in chemotherapy-treated versus vehicle-treated hTERT-BJ1 fibroblasts.

During autophagy, protein and lipid degradation occur, the latter leading to the generation of ketone bodies. A long list of up-regulated proteins involved in vesicle formation and trafficking, in protein ubiquitination pathway and proteasomal degradation, and a few enzymes involved in ketogenesis were also identified (Table 5, S3 and S4). The boost in vesicle formation and trafficking proteins could explain the greater presence of enzymes involved in fatty acid synthesis and hexosamine biosynthesis (Figure 4A and Table 1 and S2). The observed effects of taxol treatment on autophagy, mitophagy and senescence are summarised in Figure 4B.

IPA analysis confirmed an alteration of ketogenesis in fibroblasts exposed to azathioprine (Figure 5 and Table S5). IPA also showed a robust inhibitory effect of azathioprine and taxol on EIF2 signalling, responsible for protein synthesis, as measured by z score, and also revealed an alteration in the regulation of eIF4 and p70S6K signaling and in the protein ubiquitination pathway. Finally, PPARα/RXRα activation, Rho, caveolar and clathrin-mediated signalling pathways, all involved in vesicle trafficking and motility, and the mTOR signalling, known for its role in autophagy, mitochondrial metabolism and lipid metabolism, as well as cytoskeleton dynamics, were some of the top canonical pathways altered by chemotherapy, (Figure 3E, 5, and Table S5). Interestingly, p53, known mediator of senescence, was predicted to be activated in both azathioprine-treated and taxol-treated hTERT-BJ1 cells according to IPA, and TFEB, a transcriptor factor that coordinates the expression of lysosomal hydrolases, membrane proteins and genes involved in autophagy, was also predicted to be activated by taxol treatment (Figure 3F and 4C). Our previous study also reported an increased expression of p53 in hTERT-BJ1 fibroblasts treated with azatioprine and taxol by immunoblotting [11].

Thus, quantitative proteomics analysis reveals a higher presence of markers of autophagy and senescence and proteins involved in protein degradation and vesicle trafficking in hTERT-BJ1 fibroblasts, upon treatment.

#### 5 Inflammation

Senescent cells dramatically alter their secretome, enriching it with pro-inflammatory cytokines and matrix metalloproteinases. This senescence-associated secretory phenotype (SASP) can lead to chronic inflammation, which is a hallmark of aging [17]. Stromal fibroblasts secrete inflammatory cytokines when in contact with cancer cells [24]. Chemotherapy is also able to induce cytokine production in healthy tissues [12], and in particular taxol treatment induces IL6 secretion in stromal fibroblasts (Figure 1) [11]. STAT3, a known inducer of inflammation in response to stress [25, 26] was found significantly up-regulated in both azathioprine (1.69 fold increase) and taxol treatments (2.35 fold increase) relative to vehicle. STAT3 signalling was found to be activated in response to taxol also in our previous study [11].

Interestingly, several pathways involved in the inflammatory process such as IL8, acute phase response, leukocyte extravasation or Fcγ receptor-mediated phagocytosis signalling were amongst altered canonical pathways in both azathioprine-treated and taxol-treated hTERT-BJ1 cells relative to vehicle, according to IPA. N-formyl-Met-Leu-Phe (fMLP) signalling was also found to be altered in taxol-treated fibroblasts (Figure 3E and 5 and Table S5). Most of these pathways were clearly activated in taxol-treated fibroblasts as indicated by z score values, suggesting an induction of the inflammatory response in chemotherapy-treated stromal fibroblasts. Likewise, the chemokine (C-C motif) ligand 5 (CCL5 or RANTES), which plays a role in recruiting leukocytes into inflammatory sites, was identified as an upstream regulator in both treatments, particularly activated in taxol-treated fibroblasts (Figure 3F and 4C). Finally, acute phase response, which occurs soon after the onset of an inflammatory process, was identified as one of the toxic effects of both drugs (Figure 6 and Table S6). Therefore, pathway analysis of the proteomics results indicates an induction of the inflammatory response in stromal fibroblasts after exposure to chemotherapy.

### Differentially up-regulated proteins in taxol-treated fibroblasts correlate with recurrence, metastasis and poor cancer survival

**Figure 7:**
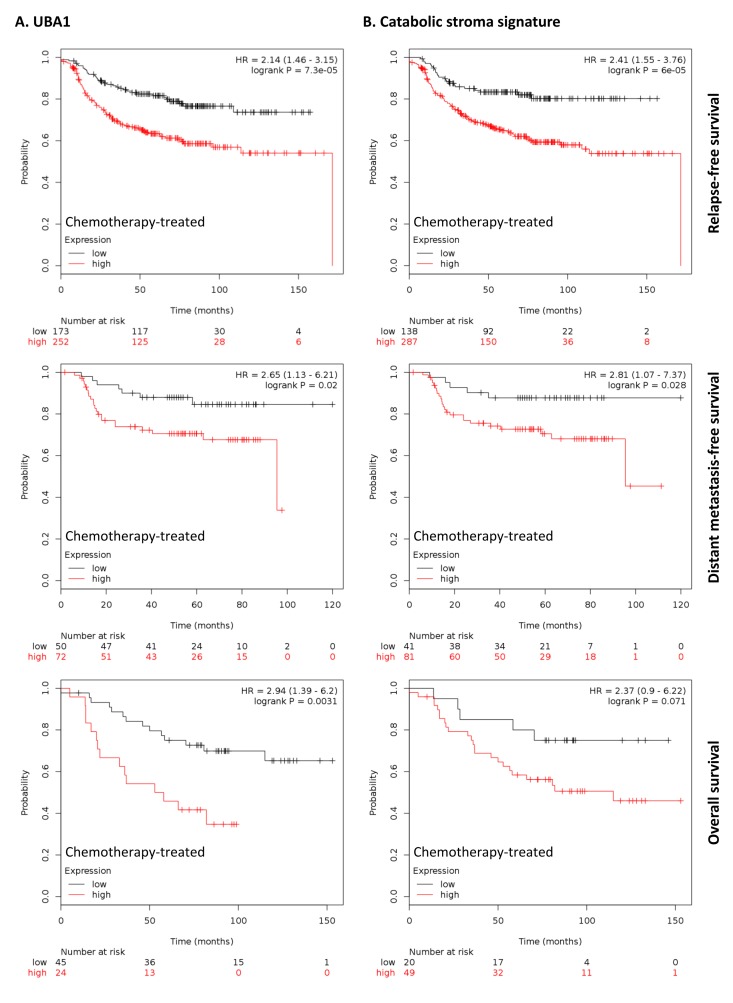
Clinical correlations of UBA1 expression and the UBA1, PSMC5, IGF2R, VAT1, HMOX1, CNN2, TLN1, GNPDA1, G6PD and FASN signature in chemotherapy-treated breast cancer patients (**A**) Correlations of UBA1 expression with relapse-free survival, distant metastasis-free survival and overall survival in breast cancer. (**B**) Correlations of the catabolic stroma signature with relapse-free survival, distant metastasis-free survival and overall survival in breast cancer. All graphs are calculated using microarray data from 425, 122 and 69 chemotherapy-treated breast cancers, respectively, determined using an online survival analysis tool. Kaplan-Meier correlations are plotted for high (above median, in red) and low (below median, in black) gene expression.

**Figure 8:**
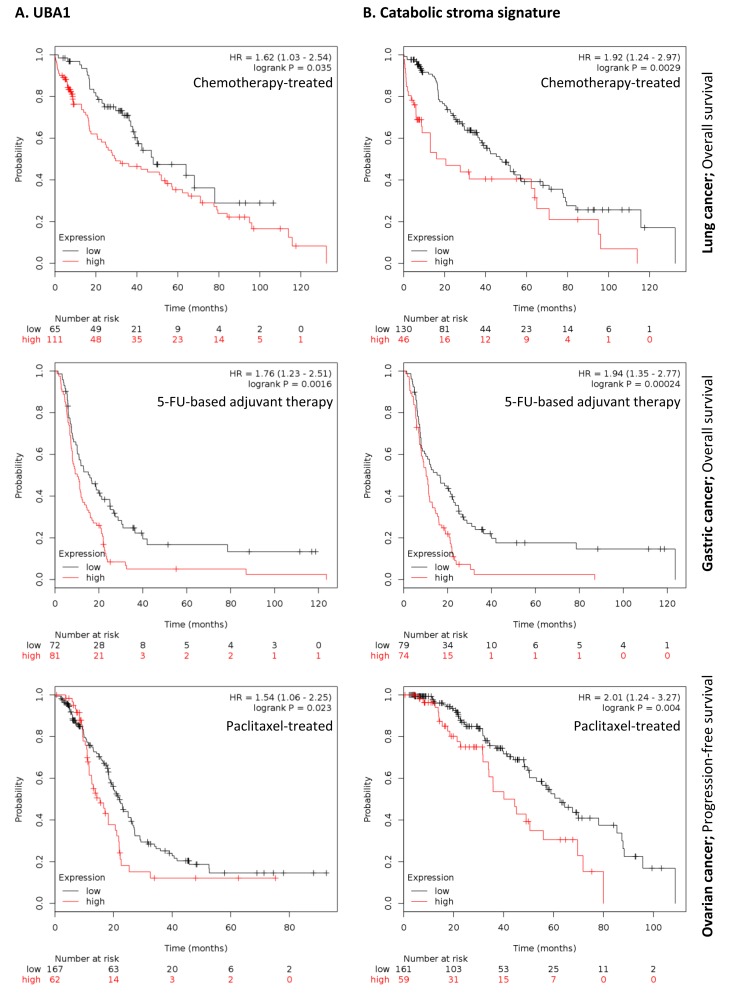
Clinical correlations of UBA1 expression and the UBA1, PSMC5, IGF2R, VAT1, HMOX1, CNN2, TLN1, GNPDA1, G6PD and FASN signature in chemotherapy-treated lung, gastric and ovarian cancer patients (**A**) Correlations of UBA1 expression with overall survival in lung and gastric cancer, and with progression-free survival in ovarian cancer. (**B**) Correlations of the catabolic stroma signature with overall survival in lung and gastric cancer, and with progression-free survival in ovarian cancer. All graphs are calculated using microarray data from 176 chemotherapy-treated lung cancers, 153 5-FU-based adjuvant therapy-treated gastric cancers, and 229 paclitaxel-treated ovarian cancers, determined using an online survival analysis tool. Kaplan-Meier correlations are plotted for high (above median, in red) and low (below median, in black) gene expression.

To further investigate the clinical implications of our proteomics datasets, we decided to test the impact of over-expressed proteins in taxol-treated hTERT-BJ1 fibroblasts in cancer prognosis. To do so, we used an on-line survival analysis tool that uses microarray gene expression data from multiple studies on breast, ovarian, lung and gastric cancer [16], which was suitable to our purpose since taxol is a chemotherapeutical drug currently used as therapy for most of these malignancies. Only those proteins with a fold change difference of 1.75 or higher and *p* values of < 0.05 (ANOVA) compared to vehicle were used for survival analyses. The expression of several proteins was found to correlate with survival in breast, ovarian, gastric and lung cancer patients. In particular, high expression of ubiquitin-like modifier activating enzyme 1 (UBA1), implicated in protein catabolism and degradation, showed a striking correlation with poor relapse-free survival, distant metastasis-free survival and overall survival in breast cancer patients previously treated with chemotherapy (425, 122 and 69 patients, respectively) (Figure 7A). The same correlation was not observed when patients who did not receive systemic treatment were considered (1000, 533 and 375 patients, respectively) (Figure S2A). Similarly, high expression of UBA1 correlated with poor overall survival in lung and gastric cancer patients previously treated with chemotherapeutic drugs (176 and 153 patients, respectively), and with progression-free survival in ovarian cancer patients particularly treated with paclitaxel (229 patients) (Figure 8A). Once more, the correlation was lost when patients who did not receive systemic treatment were considered in lung cancer (227 patients) or when patients who underwent only surgery were examined in gastric cancer (174 patients) (Figure S3A). No data from untreated ovarian cancer patients was available.

A taxol-induced catabolic stroma signature was created comprising UBA1, and other proteins representative of the catabolic CAF-like phenotype, including PSMC5, and VAT1, involved in catabolism and vesicle trafficking, several metabolic enzymes including FASN, G6PD and GNPDA, an autophagy marker, IGF2R, the oxidoreductase HMOX1, and myofibroblastic markers including CNN2 and TLN1. See Table 6 for details and abundances of these proteins in taxol-treated fibroblasts. That catabolic stroma signature showed a strong correlation with survival in treated patients (Figure 7B and 8B), whereas that correlation was not seen in non-treated patients (Figure S2B and S3B). Hence, the over-expression of proteins upon treatment with taxol is strongly linked to poor survival, treatment failure and metastasis in breast, lung, gastric and ovarian cancers. Interestingly, most markers used for the signature are also proteins found to be significantly up-regulated in azathioprine-treated hTERT-BJ1 fibroblasts relative to vehicle, including IGF2R, PSMC5, VAT1, HMOX1, CNN2, TLN1 and FASN (Tables 3, 4 5, S2, S3 and S4). Thus, we conclude that chemotherapy-mediated changes in the abundance of stromal proteins related to the CAF-like catabolic phenotype measured by quantitative proteomics associate with reduced survival, enhanced recurrence and metastasis incidence in several solid malignancies.

**Table 6:** Proteins of the catabolic stroma signature and their contribution to CAF transformation, metabolism, antioxidant response, autophagy and vesicle trafficking

CATABOLIC STROMA SIGNATURE		Taxol	Process
Ubiquitin-like modifier activating enzyme 1	UBA1	↑ 2.36	Protein degradation
26S protease regulatory subunit 8	PSMC5	↑ 3.17	Protein degradation
Cation-independent mannose-6-phosphate receptor	IGF2R	↑ 1.88	Autophagy
Synaptic vesicle membrane protein	VAT-1 VAT1	↑ 2.44	Vesicle trafficking / oxidoreductase /inhibits mitochondrial fusion
Heme oxygenase (decycling) 1	HMOX1	↑ 2.83	Antioxidant / oxidoreductase
Calponin 2	CNN2	↑ 2.76	Myofibroblastic differentiation
Talin 1	TLN1	↑ 2.95	Myofibroblastic differentiation
Glucosamine-6-phosphate deaminase 1	GNPDA1	↑ 2.46	Carbohydrate metabolism
Glucose-6-phosphate dehydrogenase	G6PD	↑ 2.69	Carbohydrate metabolism /generates antioxidant power
Fatty acid synthase	FASN	↑ 1.77	Fatty acid metabolism

## DISCUSSION

In this study we have analysed the impact of chemotherapy in the acquisition of the CAF-like, catabolic tumour stroma phenotype, which emerges in stromal fibroblasts in contact with cancer cells, and is characterised by increased glucose uptake, lactate production and extracellular acidification, increased expression of αSMA, augmented production of ROS, an activation of the JNK/AP1, HIF1, TGFβ/SMAD, STAT3 and NFkB stress-induced pathways, senescence and autophagy, and a greater secretion of inflammatory cytokines. To do so, we aimed at comprehensively characterising the cellular and metabolic changes that take place in stromal fibroblasts exposed to two common chemotherapeutic drugs: azathioprine and taxol. Label-free quantitative proteomics and extensive bioinformatics analyses revealed a protein profile characteristic for chemotherapy-treated fibroblasts that included alterations in energy metabolism, antioxidant response, autophagy and senescence, vesicle formation and trafficking, protein degradation, myofibroblastic differentiation and inflammation.

### Proteomic map of the stromal catabolic state induced by chemotherapy

The main protein set whose expression was altered in chemotherapy-treated fibroblasts was that of enzymes. Azthioprine and taxol effects on the glycolytic function of stromal fibroblasts were characterised in a previous study, which showed the induction of a glycolytic switch in hTERT-BJ1 fibroblasts via increasing their lactate production and extracellular acidification, and decreasing their ATP content [11]. We know now that this behaviour is likely to be a result of the observed up-regulation of enzymes involved in glycolysis and down-regulation of mitochondrial respiration enzymes. Indeed, our proteomic analysis shows alterations not only in glycolysis but also in pyruvate conversion to acetyl-coenzyme A and the TCA cycle. The reduction in ATP production through decreased TCA cycle activity and mitochondrial respiration may be forcing a metabolic switch that allows the cell to obtain energy from alternative metabolic processes such as glycolysis or protein catabolism. This metabolic remodelling also includes an alteration of other pathways of the carbohydrate metabolism, such as the phosphate pentose and hexosamine biosynthesis pathways, and several pathways of the fatty acid metabolism. In line with our findings, a recent study demonstrates that as an adaptive response to mitochondrial respiratory chain dysfunction and ATP deficiency, human fibroblasts up-regulate the expression of glycolytic enzymes, suggesting the induction of anaerobic glycolysis and a cellular catabolic state, in particular protein catabolism, together with autophagy [27].

According to our data, the hexosamine biosynthesis pathway is also altered in chemotherapy-treated fibroblasts, which goes in line with the increased presence of proteins involved in vesicle formation and trafficking. GNPDA1 is an enzyme of the hexosamine biosynthesis pathway, the end-products of which are used for the synthesis of membrane components such as glycolipids and proteoglycans. However, GNPDA1 catabolizes a reversible conversion between D-fructose-6-phosphate and D-glucosamine-6-phosphate, and therefore could also be used in reverse to produce substrates for glycolysis, by sacrificing structural components of the cell.

The phosphate pentose pathway is a metabolic pathway also altered by chemotherapy. During the oxidative phase of the pentose phosphate pathway, most of the reducing power of the cell is generated as NADPH. Taxol-treated fibroblasts show increased levels of the initial rate-limiting enzyme of the pentose phosphate pathway, glucose-6-phosphate dehydrogenase (G6PDH), which converts glucose-6-phosphate and NADP+ into 6-phosphoglucono-δ-lactone and NADPH. NADPH is also necessary for lipid and nucleic acid biosynthesis, and its re-oxidation to NADP+ constitutes an essential step to prevent damage by oxidative stress. Increased amounts of NADPH go in line with the upregulation of several proteins involved in antioxidant response, such as Nrf2-mediated antioxidant response proteins, as well as the greater ROS levels that we have previously seen in hTERT-BJ1 fibroblasts after taxol treatment [11]. The accumulation of ROS as a consequence of mitochondrial dysregulation during aging is also associated with DNA damage [28]. Therefore, oxidative stress and aging can be coupled in a positive feedback mechanism that accelerates cellular damage and generates a permissive metabolic microenvironment for cancer development and progression [5, 28–30].

Oxidative stress in healthy cells and tissues by most current anti-cancer therapies is thought to occur via p53 activation, which causes mitochondrial dysfunction, ROS production and downstream STAT3 signalling, promoting inflammation-related cancer [19, 26]. Our proteomics results revealed a predicted activation state of p53, mitochondrial dysfunction, increased expression of antioxidant proteins, autophagy and senescence markers, and specifically STAT3 up-regulation in chemotherapy-treated fibroblasts compared to vehicle. Our observations in vesicle trafficking and protein transport and degradation also suggest a general intensification in the transport of proteins and other cellular components to autophagosomes and lysosomes. Indeed, taxol is able to induce the formation of autophagic vesicles in stromal fibroblasts [11]. By increasing that transport, the stressed cell could be attempting to remove dysfunctional organelles such as mitochondria and other molecules that accumulate as a result of oxidative damage, or alternatively it could also be trying to retrieve molecules for re-utilisation in detriment of *de novo* protein synthesis, which is more energy consuming.

Myofibroblasts are abundant components of the reactive tumour microenvironment and are mostly accountable for the development of fibrosis [21], one of the side-effects of cancer therapy [31]. Fibroblast-to-myofibroblast differentiation increases in the stroma with age as well, resulting in an increased incidence of fibrosis-associated diseases, such as cancer [32]. Here, we report that exposure to chemotherapy can independently induce the transformation of fibroblasts into CAFs, by detecting an increase in the presence of numerous markers of myofibroblastic differentiation as measured by quantitative proteomics.

Finally, we identify several inflammation-related pathways as altered in taxol-treated fibroblasts, suggesting an induction of the inflammatory response. We previously described IL6 as one of the cytokines secreted by chemotherapy-treated fibroblasts and STAT3 target genes as being activated after treatment with taxol [11]. However, to comprehensively characterize the pro-inflammatory cytokines released by hTERT-BJ1 cells, secretome analysis should be performed for better insights.

In the present study, proteomic analyses revealed the impact of chemotherapy in the acquisition of a catabolic CAF-like phenotype in stromal fibroblasts, gradually conforming an energy-rich, pro-inflammatory microenvironment able to succour cancer cells in their battle for survival, in their purpose to metastasise, or even through which chemotherapy-damaged epithelial cells may become more susceptible to develop a fully malignant phenotype, giving rise to a new cancer. Taxol induces the catabolic stroma phenotype to a bigger extent than azathioprine. The different nature of these two drugs and the concentrations selected for the study could account for their different impact on catabolic remodelling.

### Clinical implications of the catabolic stroma signature

Quantitative proteomics allowed us to obtain a protein profile characteristic for fibroblasts exposed to azathioprine and taxol, which suggests a general alteration in their energy metabolism and a shift towards glycolysis and catabolic processes: a whole metabolic reprogramming to adapt to chemotherapy-driven mitochondrial dysfunction and oxidative stress. Likewise, the quantitative proteomics analysis allowed us to identify biomarkers of this stromal catabolic state, which were further analysed for their potential clinical implications. We generated a signature to figure out whether the chemotherapy-induced catabolic state in the stroma would have prognostic value. Indeed, the catabolic phenotype in stromal fibroblasts strikingly correlated with poor survival, treatment failure and metastatic growth in a set of breast, ovarian, lung and gastric cancer patients who were subject to chemotherapy, correlation that was lost when untreated patients were considered.

Our knowledge of the role of healthy stromal cells in metastasis and in particular in the emergence of therapy-related malignancies is still very scarce. A piece of evidence for chemotherapy-induced tumour-promoting paracrine activities of non-malignant cells has only been recently published, showing that pre-treatment of tumour-free mice with a single dose of doxorubicin is sufficient to stimulate the engraftment of lung carcinoma cells and to elevate the mitogenic activity of the serum from treated animals [12]. By label-free quantitative proteomics we detect the acquisition of a catabolic state in stromal cells due to chemotherapy-induced DNA damage, which potentially leads to the generation of an autophagic, nutrient-rich, senescent, pro-inflammatory microenvironment, the ideal niche to encourage the development of a secondary tumour or even a new carcinogenic process. However, the tumour-promoting function and the stability and reversability of the chemotherapy-induced CAF phenotype needs to be further investigated, as well as the differential expression of chemotherapy-induced catabolic stress markers on stromal fibroblasts and cancer cells *in vivo*.

To conclude, proteomic analyses revealed a significant metabolic reprogramming in response to chemotherapy. Our data provide information about novel protein targets that might enable and support different stages of the tumorigenic process, thereby opening new doors for future research. Given the essential contribution of the catabolic tumour stroma in cancer progression, it emerges as a new interesting therapeutic target. A promising approach would be the preventive inhibition of the catabolic state transformation. Indeed, TGFβ-induced myofibroblastic transformation in fibroblasts can be reversed by using antioxidants [33]. Most importantly, an antibody against fibroblast activation protein (FAP) is already being tested in clinical trials [34]. Therefore, inhibition of the catabolic stress in the tumour stroma and healthy tissues in parallel to conventional chemotherapy could help avoiding recurrence, metastasis and the growth of second primary tumours.

## MATERIALS AND METHODS

### Cell culture

All cell culture experiments were carried out using human foreskin fibroblasts immortalised with the human telomerase reverse transcriptase (hTERTBJ1 cells). hTERT-BJ1 fibroblasts were originally purchased from ATCC (CRL-4001) and maintained in DMEM media (D6546, Sigma) supplemented with 10% fetal bovine serum (FBS) (F7524, Sigma), 100 units/ml of penicillin, 100 μg/ml streptomycin (P0781, Sigma) and 1% Glutamax (#35050087, Life Technologies) at 37°C in a humidified atmosphere containing 5% CO_2_.

### Chemotherapeutical agents

Azathioprine (A4638, Sigma) and taxol or paclitaxel (Y0000698, Sigma) were used for this study at 100 μM and 100 nM, respectively.

### Sulforhodamone B (SRB) assay

SRB (S9012, Sigma) measures total biomass by staining cellular proteins. After 48 h treatment, cells were fixed in 10% trichloroacetic acid (T9159, Sigma) for 1 h at 4°C, stained with SRB (S9012, Sigma) for 15 minutes, and washed 3 times with 1% acetic acid (27225, Sigma). The incorporated die was solubilized with 10 mM Tris Base, pH 8.8 (T1503, Sigma). Absorbance was spectrophotometrically measured at 562 nm in a FluoStar Omega plate reader (BMG Labtech). Background measurements were subtracted from all values.

### Label-free quantitative proteomics

#### Chemicals and sample preparation

Formic acid, trifluoroacetic acid, ammonium formate (10 M), ammonium bicarbonate TCEP (Tris (2-carboxyethyl)phosphine hydrochloride), MMTS (Methyl methanethiosulfonate) and trypsin were all obtained from Sigma. HPLC gradient grade acetonitrile was obtained from Fisher Scientific. Briefly, 2 × 10^6^ hTERT-BJ1 fibroblasts were seeded in 150 cm plates until cells were attached. Cells were then treated with azathioprine or taxol at the concentrations indicated. As control, vehicle-treated cells were processed in parallel. After 48 hours of treatment, cells were lysed in RIPA buffer (R0278, Sigma) and kept at 4°C for 20 minutes with rotation. Lysates were cleared by centrifugation for 10 minutes at 10,000 × g and supernatants were collected and kept frozen at −80°C.

#### Protein digestion

Lysate samples were thawed to room temperature and their concentrations equalised to 1 μg/μL (50 μL volume) with RIPA buffer, and further processed for trypsin digestion by sequential reduction of disulphide bonds with TCEP and alkylation with MMTS. Briefly, 1 μL benzonase (Novagen) was added to the 50 μL aliquot and placed on ice for 15 minutes. The sample was then taken to dryness using a SpeedVac, and resuspended in 22.5 μL trypsin reaction buffer (40 mM ammonium bicarbonate and 9% acetonitrile). One μL of 50 mM TCEP solution was added to each sample, mixed briefly and placed on a heater block at 60°C for 60 minutes. After cooling to room temperature, 0.5 μL of 200 mM MMTS solution was added to each sample and allowed to react for 15 minutes. Trypsin was added in two waves to ensure efficient digestion of the sample. Firstly, 20 μg of sequencing grade trypsin was resuspended in 1800 μL of trypsin reaction buffer; 225 μL of this solution were added to each sample for digestion, and the reactions were left at 37°C overnight with shaking (600 rpm). The following morning, a further aliquot of trypsin was added. Two ml of trypsin reaction buffer was added to 20 μL of sequencing grade trypsin; 250 μL of this solution were added to each of the digest samples from overnight, and the reactions were left at 37°C for 4 hours with shaking (600 rpm). Thirty-five μL 10% formic acid were added to the 500 μL digest sample (0.7% final concentration of formic acid) to stop the digestion. The digested solution was diluted in 7.5 mL of acetonitrile containing 0.3% formic acid.

#### HILIC solid phase extraction (SPE) of peptides

PolyhydroxyethylA SPE 12 μm, 300A, 300mg cartridges (obtained from PolyLC) were used for the HILIC procedure. Prior to use, cartridges required an overnight soak in 50 mM formic acid followed by rinsing with water the following day. Cartridges were preconditioned with 2 mL of Buffer A (90% acetonitrile, 5 mM ammonium formate, pH 2.7) followed by 2 mL of Buffer B (5 mM ammonium formate, pH 2.7) and finally re-equilibrated with 10 mL Buffer A. The diluted samples were loaded onto the cartridges and washed with a further 10 mL Buffer A. Finally, peptides were eluted in 1 mL Buffer C (9 parts Buffer B plus 1 part Buffer A) and the samples dried on a Speedvac to remove organic solvent prior to LCMS/MS analysis.

#### LC-MS/MS analysis

Lyophilised digests were resuspended in 50 μL of 0.1% TFA to give an approximate concentration of 1 μg/μL. One μL injection volumes were used throughout resulting in an on-column peptide loading of approximately 1 μg per injection. Analysis was performed in quintuplicate for each sample. All LC-MS/MS analyses were performed on an LTQ Orbitrap XL mass spectrometer coupled to an Ultimate 3000 RSLCnano system (Thermo Scientific). One μL injection volumes were used throughout and samples loaded directly onto the analytical column, PepMap RSLC C18, 2 μm × 75 μm id × 50 cm (Thermo Scientific). The composition (v/v) of LC buffers were as follows; Buffer A - 99.9% water plus 0.1% formic acid and Buffer B - 80% acetonitrile, 19.9% water and 0.1% formic acid. Peptides were loaded directly onto the column at a flow rate of 400 nl/min with an initial mobile phase composition of 1% B. The organic strength was increased linearly from 1% to 22.5% B over 22.5 minutes again at 400 nl/min, followed by an increase to 24.8% B over the next 2.6 minutes with a concomitant reduction in flow rate to 300 nl/min, and to 39% B over a further 14 minutes. A further increase to 60% B over the next 5 minutes was followed by a ramp to 95% B over 2.5 minutes where it was held for a further 2 minutes. The column was then allowed to re-equilibrate to 1% B for a total analysis time of 74 minutes. The mass spectrometer was instructed to perform data dependent acquisition on the top six precursor ions, which were measured in the Orbitrap FTMS detector over the mass range 370–1200 m/z, at a nominal resolution of 60,000. MS/MS spectra were acquired in the ion trap under CID conditions with normalized collision energy of 35, isolation width of 3 Th, Q value of 0.25 and 30 ms activation time. Gasphase fractionation was performed on the five replicate injections such that MS/MS data was collected for precursor ion range 370–494 m/z Injection 1, 494–595 m/z Injection 2, 595–685 m/z Injection 3, 685–817 m/z Injection 4 and 817–1200 m/z Injection 5.

#### Statistical analysis

Xcalibur raw data files acquired on the LTQ-Orbitrap XL were directly imported into Progenesis LCMS software (Waters Corp) for peak detection and alignment. Data were analysed using the Mascot search engine. Five replicates were analysed for each sample type (N = 5). Statistical analyses were performed using ANOVA and only fold-changes in proteins with a *p*-value less than 0.05 were considered significant.

### Ingenuity pathway analyses

Pathway and function analyses were generated using Ingenuity Pathway Analysis (IPA) (Ingenuity systems, http://www.ingenuity.com), which assists with proteomics data interpretation via grouping differentially expressed genes or proteins into known functions and pathways. Pathways with a z score > 1.9 were considered as significantly activated, and pathways with a z score < −1.9 were considered as significantly inhibited.

### Graphs and correlation analyses

All graphs were done in Microsoft Excel except for correlation graphs. Correlations between protein expression and patient survival were calculated using a survival analysis tool available online (http://kmplot.com) [16].

## SUPPLEMENTARY INFORMATION FIGURES AND TABLES







## Abbreviations

AZA: azathioprine
TAX: taxol
CAF: cancer-associated fibroblast
MCT4: monocarboxylate transporter 4
IL6: interleukin 6
ROS: reactive oxygen species
αSMA: α-smooth muscle actin

## References

[1] Elkhattouti A, Hassan M, Gomez CR (2015). Stromal Fibroblast in Age-Related Cancer: Role in Tumorigenesis and Potential as Novel Therapeutic Target. Frontiers in oncology.

[2] Lasry A, Ben-Neriah Y (2015). Senescence-associated inflammatory responses: aging and cancer perspectives. Trends in immunology.

[3] Hanahan D, Coussens LM (2012). Accessories to the crime: functions of cells recruited to the tumor microenvironment. Cancer Cell.

[4] Lisanti MP, Sotgia F, Pestell RG, Howell A, Martinez-Outschoorn UE (2013). Stromal glycolysis and MCT4 are hallmarks of DCIS progression to invasive breast cancer. Cell Cycle.

[5] Balliet RM, Capparelli C, Guido C, Pestell TG, Martinez-Outschoorn UE, Lin Z, Whitaker-Menezes D, Chiavarina B, Pestell RG, Howell A, Sotgia F, Lisanti MP (2011). Mitochondrial oxidative stress in cancer-associated fibroblasts drives lactate production, promoting breast cancer tumor growth: understanding the aging and cancer connection. Cell Cycle.

[6] Bartling B, Hofmann HS, Silber RE, Simm A (2008). Differential impact of fibroblasts on the efficient cell death of lung cancer cells induced by paclitaxel and cisplatin. Cancer Biol Ther.

[7] Ertel A, Tsirigos A, Whitaker-Menezes D, Birbe RC, Pavlides S, Martinez-Outschoorn UE, Pestell RG, Howell A, Sotgia F, Lisanti MP (2012). Is cancer a metabolic rebellion against host aging? In the quest for immortality, tumor cells try to save themselves by boosting mitochondrial metabolism. Cell Cycle.

[8] Nieman KM, Kenny HA, Penicka CV, Ladanyi A, Buell-Gutbrod R, Zillhardt MR, Romero IL, Carey MS, Mills GB, Hotamisligil GS, Yamada SD, Peter ME, Gwin K, Lengyel E (2011). Adipocytes promote ovarian cancer metastasis and provide energy for rapid tumor growth. Nat Med.

[9] Zhang W, Trachootham D, Liu J, Chen G, Pelicano H, Garcia-Prieto C, Lu W, Burger JA, Croce CM, Plunkett W, Keating MJ, Huang P (2012). Stromal control of cystine metabolism promotes cancer cell survival in chronic lymphocytic leukaemia. Nat Cell Biol.

[10] Sun Y, Campisi J, Higano C, Beer TM, Porter P, Coleman I, True L, Nelson PS (2012). Treatment-induced damage to the tumor microenvironment promotes prostate cancer therapy resistance through WNT16B. Nature medicine.

[11] Peiris-Pagès MS, Sotgia F, Lisanti MP (2015). Chemotherapy induces the cancer-associated fibroblast phenotype, activating paracrine Hedgehog-GLI signaling in breast cancer cells. Oncotarget.

[12] Porter DC, Farmaki E, Altilia S, Schools GP, West DK, Chen M, Chang BD, Puzyrev AT, Lim CU, Rokow-Kittell R, Friedhoff LT, Papavassiliou AG, Kalurupalle S, Hurteau G, Shi J, Baran PS (2012). Cyclin-dependent kinase 8 mediates chemotherapy-induced tumor-promoting paracrine activities. Proc Natl Acad Sci U S A.

[13] Karran P, Attard N (2008). Thiopurines in current medical practice: molecular mechanisms and contributions to therapy-related cancer. Nat Rev Cancer.

[14] Raja FA, Chopra N, Ledermann JA (2012). Optimal first-line treatment in ovarian cancer. Annals of oncology : official journal of the European Society for Medical Oncology / ESMO.

[15] Vyas D, Laput G, Vyas AK (2014). Chemotherapy-enhanced inflammation may lead to the failure of therapy and metastasis. OncoTargets and therapy.

[16] Gyorffy B, Lanczky A, Eklund AC, Denkert C, Budczies J, Li Q, Szallasi Z (2010). An online survival analysis tool to rapidly assess the effect of 22,277 genes on breast cancer prognosis using microarray data of 1,809 patients. Breast cancer research and treatment.

[17] Lopez-Otin C, Blasco MA, Partridge L, Serrano M, Kroemer G (2013). The hallmarks of aging. Cell.

[18] Kumari KK, Setty OH (2012). Protective effect of Phyllanthus fraternus against mitochondrial dysfunction induced by coadministration of cisplatin and cyclophosphamide. J Bioenerg Biomembr.

[19] Velez JM, Miriyala S, Nithipongvanitch R, Noel T, Plabplueng CD, Oberley T, Jungsuwadee P, Van Remmen H, Vore M, St Clair DK (2011). p53 Regulates oxidative stress-mediated retrograde signaling: a novel mechanism for chemotherapy-induced cardiac injury. PLoS One.

[20] Paunescu V, Bojin FM, Tatu CA, Gavriliuc OI, Rosca A, Gruia AT, Tanasie G, Bunu C, Crisnic D, Gherghiceanu M, Tatu FR, Tatu CS, Vermesan S (2011). Tumour-associated fibroblasts and mesenchymal stem cells: more similarities than differences. J Cell Mol Med.

[21] Desmouliere A, Darby IA, Gabbiani G (2003). Normal and pathologic soft tissue remodeling: role of the myofibroblast, with special emphasis on liver and kidney fibrosis. Lab Invest.

[22] Campisi J, d'Adda di Fagagna F (2007). Cellular senescence: when bad things happen to good cells. Nature reviews Molecular cell biology.

[23] Goehe RW, Di X, Sharma K, Bristol ML, Henderson SC, Valerie K, Rodier F, Davalos AR, Gewirtz DA (2012). The autophagy-senescence connection in chemotherapy: must tumor cells (self) eat before they sleep?. J Pharmacol Exp Ther.

[24] Martinez-Outschoorn UE, Whitaker-Menezes D, Lin Z, Flomenberg N, Howell A, Pestell RG, Lisanti MP, Sotgia F (2011). Cytokine production and inflammation drive autophagy in the tumor microenvironment: role of stromal caveolin-1 as a key regulator. Cell Cycle.

[25] Grivennikov SI, Greten FR, Karin M (2010). Immunity, inflammation, and cancer. Cell.

[26] Kamp DW, Shacter E, Weitzman SA (2011). Chronic inflammation and cancer: the role of the mitochondria. Oncology.

[27] Marin-Buera L, Garcia-Bartolome A, Moran M, Lopez-Bernardo E, Cadenas S, Hidalgo B, Sanchez R, Seneca S, Arenas J, Martin MA, Ugalde C (2015). Differential proteomic profiling unveils new molecular mechanisms associated with mitochondrial complex III deficiency. Journal of proteomics.

[28] Lisanti MP, Martinez-Outschoorn UE, Pavlides S, Whitaker-Menezes D, Pestell RG, Howell A, Sotgia F (2011). Accelerated aging in the tumor microenvironment: connecting aging, inflammation and cancer metabolism with personalized medicine. Cell cycle.

[29] Martinez-Outschoorn UE, Curry JM, Ko YH, Lin Z, Tuluc M, Cognetti D, Birbe RC, Pribitkin E, Bombonati A, Pestell RG, Howell A, Sotgia F, Lisanti MP (2013). Oncogenes and inflammation rewire host energy metabolism in the tumor microenvironment: RAS and NFkappaB target stromal MCT4. Cell Cycle.

[30] Lisanti MP, Martinez-Outschoorn UE, Lin Z, Pavlides S, Whitaker-Menezes D, Pestell RG, Howell A, Sotgia F (2011). Hydrogen peroxide fuels aging, inflammation, cancer metabolism and metastasis: the seed and soil also needs “fertilizer”. Cell cycle.

[31] Salata C, Ferreira-Machado SC, De Andrade CB, Mencalha AL, Mandarim-De-Lacerda CA, de Almeida CE (2014). Apoptosis induction of cardiomyocytes and subsequent fibrosis after irradiation and neoadjuvant chemotherapy. International journal of radiation biology.

[32] Sampson N, Berger P, Zenzmaier C (2012). Therapeutic targeting of redox signaling in myofibroblast differentiation and age-related fibrotic disease. Oxidative medicine and cellular longevity.

[33] Cat B, Stuhlmann D, Steinbrenner H, Alili L, Holtkotter O, Sies H, Brenneisen P (2006). Enhancement of tumor invasion depends on transdifferentiation of skin fibroblasts mediated by reactive oxygen species. Journal of cell science.

[34] Scott AM, Wiseman G, Welt S, Adjei A, Lee FT, Hopkins W, Divgi CR, Hanson LH, Mitchell P, Gansen DN, Larson SM, Ingle JN, Hoffman EW, Tanswell P, Ritter G, Cohen LS (2003). A Phase I dose-escalation study of sibrotuzumab in patients with advanced or metastatic fibroblast activation protein-positive cancer. Clinical cancer research: an official journal of the American Association for Cancer Research.

