# Supplementary material for: Proteomic identification of prognostic tumour biomarkers, using chemotherapy-induced cancer-associated fibroblasts
Source: Aging (Albany NY). 2015 Oct 23;7(10):816–38. doi: 10.18632/aging.100808 (PMC4637208; doi:10.18632/aging.100808)
Supplement: Supplementary file 1 [file aging-07-816-s001.docx]

**SUPPLEMENTARY FIGURE LEGENDS**

**Supplementary Figure 1. The concentrations of azathioprine and taxol selected for the current study were sub-lethal after 48 h treatment**

**A)** Representative pictures of hTERT-BJ1 fibroblasts treated for 48 h with either azathioprine or taxol. Vehicle-treated and untreated cells are also shown

**B)** SRB staining of hTERT-BJ1 fibroblasts after 48 h treatment with 100 uM of azathioprine and 100 nM of taxol relative to vehicle-treated cells. In both cases, over 60% of cells remained viable

**Supplementary Figure 2. Clinical correlations of UBA1 expression and the UBA1, PSMC5, IGF2R, VAT1, HMOX1, CNN2, TLN1, GNPDA1, G6PD and FASN signature in systemically untreated breast cancer patients.**

**A)** Correlations of UBA1 expression with relapse-free survival, distant metastasis-free survival and overall survival in breast cancer.

**B)** Correlations of the catabolic stroma signature with relapse-free survival, distant metastasis-free survival and overall survival in breast cancer. All graphs are calculated using microarray data from 1000, 533 and 375 systemically untreated breast cancers, respectively, determined using an online survival analysis tool. Kaplan-Meier correlations are plotted for high (above median, in red) and low (below median, in black) gene expression, and show loss of correlation or less significance in untreated patients.

**Supplementary Figure 3. Clinical correlations of UBA1 expression and the UBA1, PSMC5, IGF2R, VAT1, HMOX1, CNN2, TLN1, GNPDA1, G6PD and FASN signature systemically untreated lung and gastric cancer patients.**

**A)** Correlations of UBA1 expression with overall survival in lung and gastric cancer, and with progression-free survival in ovarian cancer.

**B)** Correlations of the catabolic stroma signature with overall survival in lung and gastric cancer, and with progression-free survival in ovarian cancer. All graphs are calculated using microarray data from 227 untreated lung cancers, and 174 gastric cancer patients who underwent surgery, determined using an online survival analysis tool. Kaplan-Meier correlations are plotted for high (above median, in red) and low (below median, in black) gene expression, and show loss of correlation or less significance in untreated patients.

**SUPPLEMENTARY TABLE LEGENDS**

**Supplementary Table 1. Changes in the expression of proteins involved in mitochondrial function after treatment with azathioprine and taxol for 48 h, as measured by quantitative proteomics and indicated by IPA analysis.** Taxol treatment remarkably decreased the expression of proteins representative of mitochondrial activity. Proteins that show increased expression after treatment are shown in red, and proteins that are decreased are shown in green. Number indicates the fold increase or fold decrease in protein expression in chemotherapy-treated versus vehicle-treated hTERT-BJ1 fibroblasts.

**Supplementary Table 2. Changes in the expression of enzymes involved in lipid metabolism after treatment with azathioprine and taxol for 48 h, as measured by quantitative proteomics.** Taxol treatment mainly decreased the expression of enzymes of mitochondrial β-oxidation and enhanced that of lipid biosynthesis. Enzymes that show increased expression after treatment are shown in red, and enzymes that are decreased are shown in green. Number indicates the fold increase or fold decrease in protein expression in chemotherapy-treated versus vehicle-treated hTERT-BJ1 fibroblasts.

**Supplementary Table 3. Changes in the expression of proteins involved in vesicle formation and trafficking after treatment with azathioprine and taxol for 48 h as measured by quantitative proteomics.** Chemotherapy enormously increased the expression of proteins involved vesicle formation and trafficking. Proteins that show amplified expression after treatment are shown in red, and proteins that are decreased are shown in green. Number indicates the fold increase or fold decrease in protein expression in chemotherapy-treated versus vehicle-treated hTERT-BJ1 fibroblasts.

**Supplementary Table 4. Changes in the expression of proteins involved in protein transport, ubiquitination, and proteasomal degradation after treatment with azathioprine and taxol for 48 h as measured by quantitative proteomics.** Proteins that show amplified expression after treatment are shown in red, and proteins that are decreased are shown in green. Number indicates the fold increase or fold decrease in protein expression in chemotherapy-treated versus vehicle-treated hTERT-BJ1 fibroblasts.

**Supplementary Table 5. Pathway analysis of differentially expressed proteins in hTERT-BJ1 fibroblasts treated with azathioprine or taxol compared to vehicle-treated cells.**

**A)** Ingenuity Pathway Analysis showed canonical pathways significantly altered by the proteins differentially expressed in hTERT-BJ1 fibroblasts treated with azathioprine or taxol (P < 0.05). The p value, the z score, and the ratio of the number of proteins differentially expressed in a given pathway divided by the total number of proteins that make up that pathway for each pathway are indicated.

**Supplementary Table 6. Toxicity effects of differentially expressed proteins in hTERT-BJ1 fibroblasts treated with azathioprine or taxol compared to vehicle-treated cells.**

**A)** Ingenuity Pathway Analysis showed toxicity functions significantly enriched by the proteins differentially expressed in hTERT-BJ1 fibroblasts treated with azathioprine or taxol (P < 0.05). The p value and the ratio of the number of proteins differentially expressed in a given pathway divided by the total number of proteins that make up that pathway for each pathway are indicated.
