# Supplementary material for: Proteomic identification of prognostic tumour biomarkers, using chemotherapy-induced cancer-associated fibroblasts
Source: Aging (Albany NY). 2015 Oct 23;7(10):816–38. doi: 10.18632/aging.100808 (PMC4637208; doi:10.18632/aging.100808)

Figure S1

A **48h treatment**

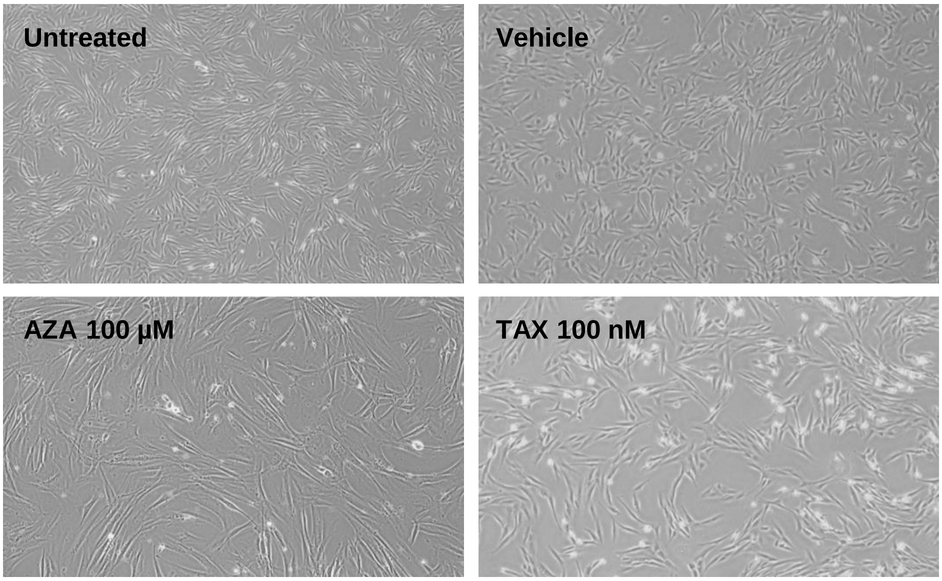

B

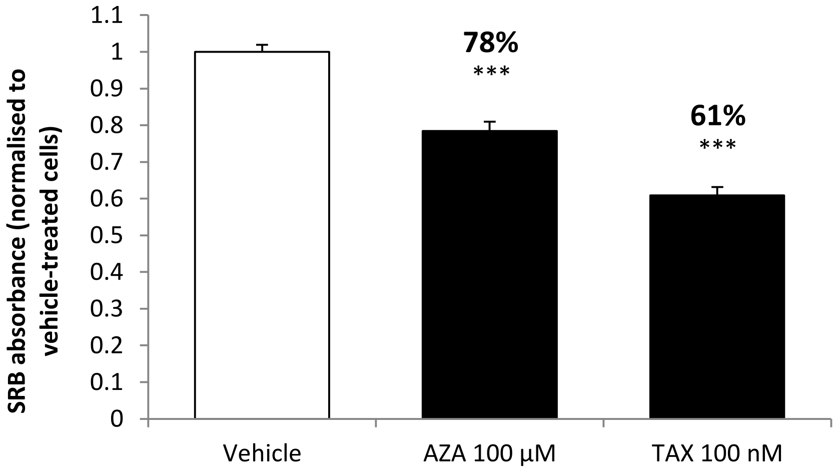

Figure S2

A. UBA1 **B. Catabolic stroma signature**

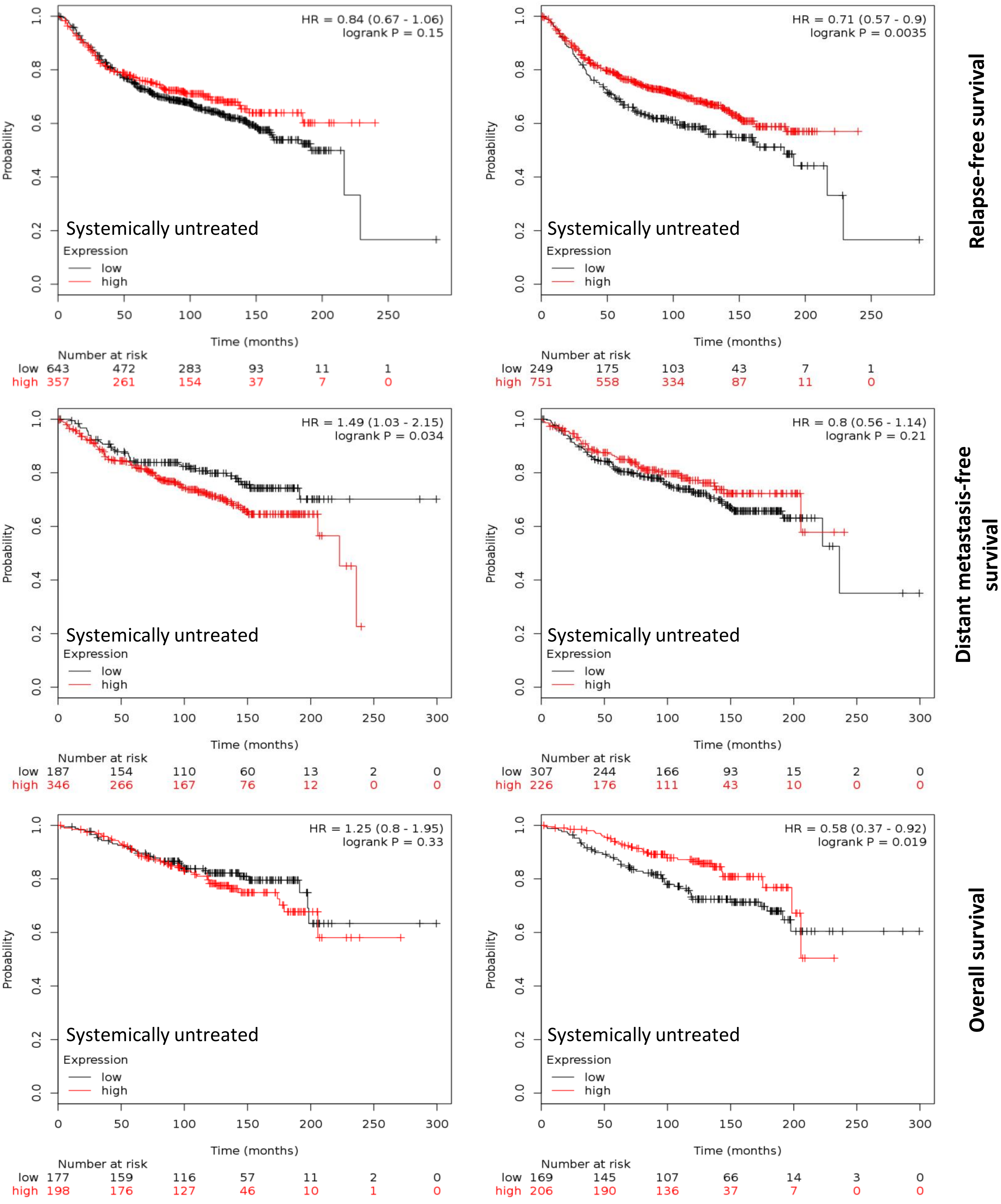

Figure S3

A. UBA1 **B. Catabolic stroma signature**

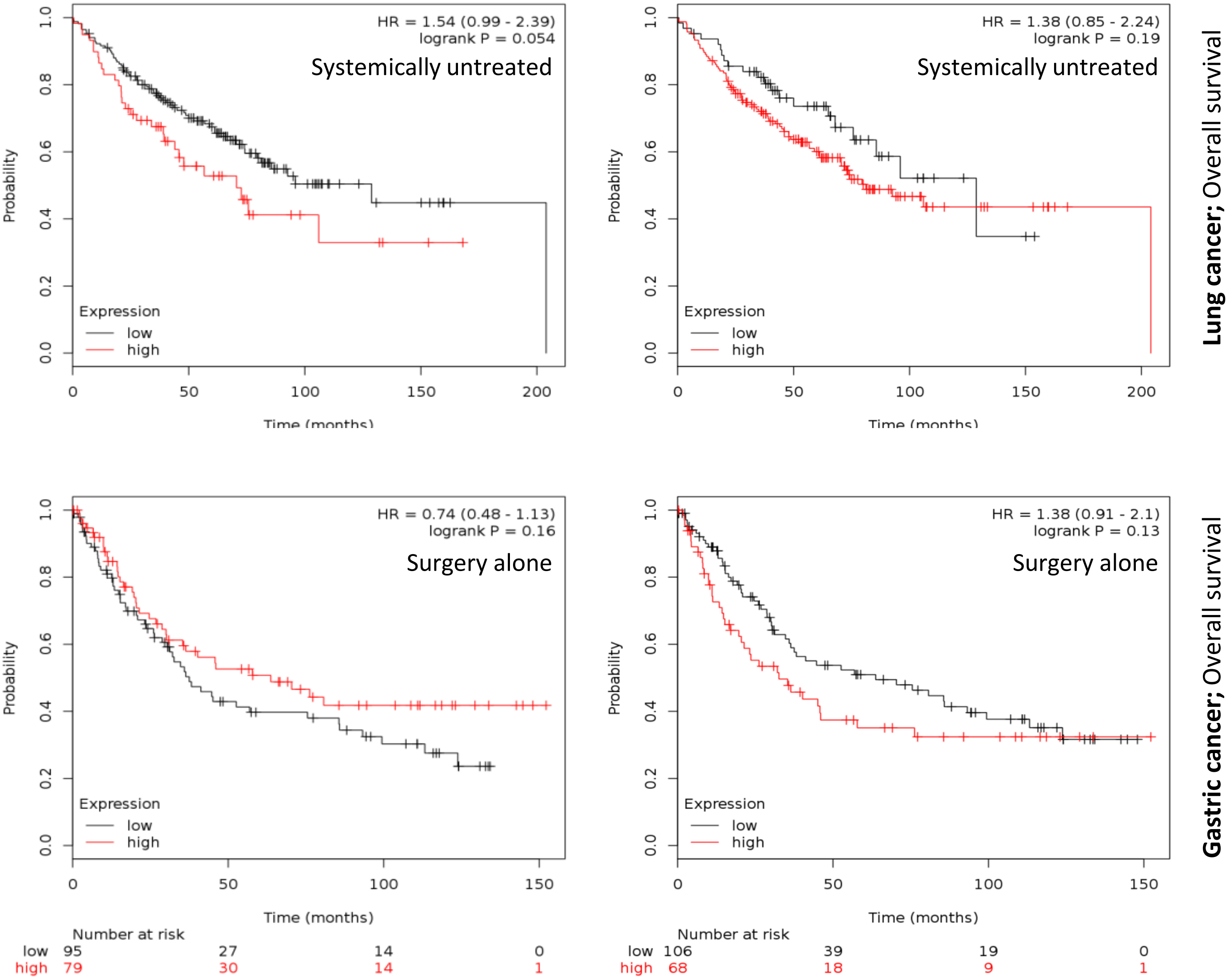

Supplement: Supplementary file 2 [file aging-07-816-s002.pdf]
