# Supplementary material for: Proteomic identification of prognostic tumour biomarkers, using chemotherapy-induced cancer-associated fibroblasts
Source: Aging (Albany NY). 2015 Oct 23;7(10):816–38. doi: 10.18632/aging.100808 (PMC4637208; doi:10.18632/aging.100808)
Supplement: Supplementary file 3 [file aging-07-816-s003.docx]

**Table S1**

| **MITOCHONDRIAL DYSFUNCTION** | | **Azathioprine** | **Taxol** |
| --- | --- | --- | --- |
| Aconitase 2, mitochondrial | ACO2 | ↑ 1.41 | ↓ 1.23 |
| Apoptosis-inducing factor, mitochondrion-associated 1 | AIFM1 |  | ↑ 1.74 |
| Amyloid beta (A4) precursor protein | APP | ↑ 2.57 |  |
| ATP synthase, mitochondrial | ATP5A1 | ↓ 1.51 | ↓ 1.16 |
|  | ATP5B |  | ↑ 2.36 |
|  | ATP5F1 | ↓ 1.70 | ↓ 1.51 |
|  | ATP5H | ↑ 1.56 |  |
|  | ATP5J | ↑ 1.36 |  |
| COX17 cytochrome c oxidase copper chaperone | COX17 |  | ↓ 2.00 |
| Cytochrome c oxidase | COX5A |  | ↓ 1.39 |
|  | COX5B | ↑ 1.37 |  |
|  | COX6A1 | ↑ 1.47 |  |
|  | COX6C |  | ↓ 1.62 |
| Cytochrome b5 reductase 3 | CYB5R3 | ↑ 1.52 | ↑ 1.36 |
| Cytochrome c | CYCS |  | ↓ 1.61 |
| Glycerol-3-phosphate dehydrogenase 2, mitochondrial | GPD2 | ↓ 1.51 | ↓ 1.33 |
| Hydroxysteroid (17-beta) dehydrogenase, mitochondrial | HSD17B10 |  | ↓ 1.62 |
| NADH dehydrogenase (ubiquinone) | NDUFB10 |  | ↓ 1.29 |
|  | NDUFS1 |  | ↓ 1.73 |
|  | NDUFS7 |  | ↓ 1.31 |
|  | NDUFV1 |  | ↑ 1.54 |
|  | NDUFV2 |  | ↓ 1.45 |
| 2-oxoglutarate dehydrogenase | OGDH | ↓ 1.37 |  |
| Parkinson protein 7 | PARK7 | ↑ 1.80 |  |
| Peroxiredoxin 5 | PRDX5 |  | ↑ 1.33 |
| Succinate dehydrogenase complex | SDHA |  | ↓ 2.43 |
|  | SDHB | ↓ 1.51 |  |
| Superoxide dismutase 2, mitochondrial | SOD2 | ↑ 1.69 | ↓ 1.87 |
| Ubiquinol-cytochrome c reductase | UQCRB |  | ↑ 1.62 |
|  | UQCRC1 |  | ↓ 1.42 |
| Voltage-dependent anion | VDAC1 | ↑ 1.54 |  |
|  | VDAC2 | ↑ 1.32 |  |

**Table S2**

| **MITOCHONDRIAL FATTY ACID BETA-OXIDATION** | | **Azathioprine** | **Taxol** |
| --- | --- | --- | --- |
| Acyl-CoA dehydrogenase | ACADM |  | ↓ 7.73 |
|  | ACADVL | ↑ 1.51 | ↑ 1.29 |
| Enoyl-CoA hydratase | HSD17B10 |  | ↓ 1.62 |
| Enoyl-CoA delta isomerase 2 | ECI2 |  | ↓ 1.34 |
| Hydroxyacyl-CoA dehydrogenase | HADH |  | ↓ 1.43 |
|  | HADHA | ↓ 1.57 |  |
|  | HADHB | ↑ 1.36 |  |
| **PEROXISOMAL FATTY ACID BETA-OXIDATION** | | **Azathioprine** | **Taxol** |
| Acyl-CoA acyltransferase 1 | ACAA1 | ↑ 1.26 |  |
| Enoyl-CoA hydratase | HSD17B4 |  | ↑ 1.82 |
| **FATTY ACID BIOSYNTHESIS** | | **Azathioprine** | **Taxol** |
| ATP citrate lyase | ACLY |  | ↑ 2.07 |
| Fatty acid synthase | FASN | ↑ 2.42 | ↑ 1.77 |
| Fatty acid desaturase 2 | FADS2 |  | ↓ 3.04 |
| Acyl-CoA synthetase long-chain family member | ACSL1 |  | ↑ 2.21 |
|  | ACSL3 |  | ↑ 1.56 |
|  | ACSL4 |  | ↓ 1.62 |
| Acyl-CoA thioesterase | ACOT9 |  | ↑ 1.59 |
| Elongation of very long chain fatty acids protein 1 | ELOVL1 |  | ↓ 1.36 |

**Table S3**

| **Vesicle Formation and Trafficking** | | **Azathioprine** | **Taxol** |
| --- | --- | --- | --- |
| Beta-centractin | ACTR1B | ↑ 3.28 | ↑ 1.50 |
| Caveolin 1 | CAV1 | ↑ 1.49 | ↑ 2.05 |
| Clathrin | CLTA | ↑ 1.34 | ↑ 1.44 |
|  | CLTB |  | ↑ 1.46 |
|  | CLTC | ↓ 1.80 | ↑ 1.90 |
|  | CLTCL1 | ↑ 2.06 |  |
| Clathrin interactor 1 | CLINT1 |  | ↑ 2.09 |
| Coatomer protein complex | COPB1 |  | ↑ 2.11 |
|  | COPB2 |  | ↑ 1.22 |
|  | COPA | ↑ 1.74 | ↓ 1.64 |
| Cortactin | CTTN | ↑ 1.80 | ↑ 2.12 |
| Dipeptidyl peptidase 4 | DPP4 |  | ↑ 3.05 |
| Dynactin 2 | DCTN2 |  | ↑ 1.63 |
| ELKS/Rab6-interacting/CAST family member 1 | ERC1 | ↑ 1.55 | ↑ 2.22 |
| General vesicular transport factor p115 | USO1 | ↑ 1.16 | ↑ 1.64 |
| Golgin | GOLGB1 | ↑ 1.43 | ↑ 1.63 |
|  | GOLGA3 |  | ↑ 1.52 |
| Phosphatidylinositol binding clathrin assembly protein | PICALM |  | ↓ 3.37 |
| Prolow-density lipoprotein receptor-related protein 1 | LRP1 |  | ↑ 1.91 |
| Protein transport proteins | SEC22B | ↑ 1.64 |  |
|  | SEC23A |  | ↓ 1.72 |
|  | SEC23B | ↑ 2.47 | ↑ 1.77 |
|  | SEC24D |  | ↑ 1.46 |
|  | SEC31A |  | ↑ 2.70 |
|  | SEC61A1 |  | ↓ 1.31 |
|  | SEC61A2 | ↑ 1.27 |  |
|  | SEC61B |  | ↓ 2.39 |
| Ras-related protein | RAB5C |  | ↓ 1.38 |
|  | RAB7A | ↑ 1.31 |  |
| Secretory carrier-associated membrane protein 4 | SCAMP4 |  | ↑ 1.34 |
| Sorting nexins | SNX3 |  | ↑ 1.78 |
|  | SNX9 |  | ↑ 3.15 |
|  | SNX18 |  | ↑ 2.85 |
| Synaptic vesicle membrane protein VAT-1 | VAT1 | ↑ 2.01 | ↑ 2.44 |
| Transferrin receptor | TFRC | ↑ 2.18 |  |
| Vacuolar protein sorting-associated protein | VPS29 |  | ↑ 2.29 |
|  | VPS13B | ↑ 1.52 |  |
|  | VPS35 |  | ↑ 1.45 |

**Table S4**

| **Protein Transport and Ubiquitination** | | **Azathioprine** | **Taxol** |
| --- | --- | --- | --- |
| HECT, UBA and WWE domain containing 1, E3 Ubiquitin protein ligase | HUWE | ↓ 1.94 | ↓ 1.58 |
| MYC binding protein 2, E3 ubiquitin protein ligase | MYCBP2 |  | ↑ 2.07 |
| Ubiquitin-like modifier activating enzyme | UBA1 | ↓ 2.10 | ↑ 2.36 |
|  | UBA3 | ↓ 1.75 |  |
| Ubiquitin associated protein 2-like | UBAP2L | ↓ 1.46 |  |
| Ubiquitin carboxyl-terminal esterase L1 (ubiquitin thiolesterase) | UCHL1 |  | ↑ 1.71 |
| Ubiquitin specific peptidase | USP5 | ↓ 2.11 |  |
|  | USP33 | ↑ 1.82 |  |
| Heat shock protein 90kDa | HSP90AA1 | ↓ 1.67 |  |
|  | HSP90AB1 | ↓ 2.16 | ↓ 1.46 |
|  | HSP90B1 | ↑ 1.54 | ↓ 1.88 |
| Heat shock 70kDa protein | HSPA1A/B |  | ↑ 1.67 |
|  | HSPA2 |  | ↓ 2.47 |
|  | HSPA4 | ↓ 1.88 |  |
|  | HSPA5 | ↑ 1.40 | ↑ 1.71 |
|  | HSPA6 |  | ↑ 1.50 |
|  | HSPA8 | ↓ 1.26 |  |
|  | HSPA9 | ↑ 1.92 | ↓ 1.47 |
| Heat shock 27kDa protein 1 | HSPB1 |  | ↑ 1.90 |
| Heat shock 60kDa protein 1 (chaperonin) | HSPD1 |  | ↓ 1.97 |
| Heat shock 10kDa protein 1 | HSPE1 |  | ↓ 1.65 |
| Heat shock protein 90kDa | HSP90AA1 | ↓ 1.67 |  |
|  | HSP90AB1 | ↓ 2.16 | ↓ 1.46 |
|  | HSP90B1 | ↑ 1.54 | ↓ 1.88 |
| Major histocompatibility complex, class I | HLA-A | ↑ 1.31 |  |
|  | HLA-B | ↑ 1.77 | ↑ 1.74 |
|  | HLA-C |  | ↓ 1.87 |
| USO1 vesicle transport factor | USO1 | ↑ 1.16 | ↑ 1.64 |
| **Proteasomal Degradation** | | **Azathioprine** | **Taxol** |
| 26S proteasome subunit alpha | PSMA7 | ↓ 1.35 |  |
| 26S proteasome non-ATPase regulatory subunit | PSMD2 | ↑ 1.47 | ↑ 1.33 |
|  | PSMD4 |  | ↑ 4.56 |
|  | PSMD7 | ↓ 1.38 |  |
|  | PSMD11 |  | ↑ 1.38 |
| 26S protease regulatory subunit | PSMC2 | ↓2.08 | ↓1.43 |
|  | PSMC3 |  | ↑ 1.45 |
|  | PSMC4 |  | ↓ 1.93 |
|  | PSMC5 | ↑ 2.12 | ↑ 3.17 |
|  | PSMC6 |  | ↓1.43 |
| Proteasome activator complex subunit 1 | PSME1 |  | ↑ 1.47 |
|  | PSME2 |  | ↓2.26 |
|  | PSME3 | ↓2.04 |  |

**Table S5**

| **Canonical Pathways** | **Azathioprine 100 µM** | | | **Taxol 100 nM** | | |
| --- | --- | --- | --- | --- | --- | --- |
|  | p-value | z score | Ratio | p-value | z score | Ratio |
| EIF2 Signaling | 2.5E-45 | -3.138 | 0.36 | 6.3E-57 | -5.692 | 0.45 |
| Regulation of eIF4 and p70S6K Signaling | 1.3E-24 | 0.000 | 0.28 | 5.0E-23 | 0.000 | 0.30 |
| mTOR Signaling | 1.6E-18 |  | 0.20 | 2.5E-15 |  | 0.21 |
| Actin Cytoskeleton Signaling | 1.7E-08 |  | 0.12 | 6.3E-14 |  | 0.19 |
| Caveolar-mediated Endocytosis Signaling | 2.2E-09 |  | 0.22 | 5.0E-13 |  | 0.31 |
| Remodeling of Epithelial Adherens Junctions | 5.0E-12 |  | 0.27 | 7.9E-12 | 1.890 | 0.30 |
| Epithelial Adherens Junction Signaling | 3.9E-09 |  | 0.15 | 1.0E-11 |  | 0.20 |
| Integrin Signaling | 4.7E-05 | 2.183 | 0.10 | 3.0E-10 | 3.157 | 0.16 |
| Regulation of Actin-based Motility by Rho | 1.9E-04 | 1.265 | 0.13 | 1.7E-09 | 2.524 | 0.23 |
| Virus Entry via Endocytic Pathways | 5.6E-08 |  | 0.18 | 2.6E-09 |  | 0.23 |
| RhoA Signaling | 8.9E-04 | 0.905 | 0.10 | 2.3E-08 | 2.837 | 0.18 |
| Calcium Signaling | 2.5E-05 | 2.449 | 0.11 | 6.2E-08 | -0.707 | 0.15 |
| Signaling by Rho Family GTPases | 4.8E-04 | 2.138 | 0.08 | 9.3E-08 | 3.400 | 0.13 |
| Granzyme A Signaling | 1.5E-05 |  | 0.35 | 3.2E-07 |  | 0.47 |
| Cdc42 Signaling | 1.7E-03 |  | 0.09 | 3.9E-07 |  | 0.16 |
| ILK Signaling | 1.5E-03 | 2.673 | 0.08 | 8.1E-07 | 1.706 | 0.14 |
| Protein Kinase A Signaling | 1.0E-03 |  | 0.07 | 2.6E-06 |  | 0.10 |
| TCA Cycle II (Eukaryotic) | 7.4E-03 |  | 0.17 | 4.9E-06 |  | 0.35 |
| Mitochondrial Dysfunction | 1.9E-04 |  | 0.10 | 6.5E-06 |  | 0.13 |
| Leukocyte Extravasation Signaling | 1.1E-03 | 0.277 | 0.08 | 8.5E-06 | 1.964 | 0.12 |
| PPARa/RXRa activation | 1.2E-02 | -1.342 | 0.073 | 4.0E-02 | 1.134 | 0.08 |
| Clathrin-mediated Endocytosis Signaling | 2.6E-02 |  | 0.07 | 1.2E-05 |  | 0.13 |
| RhoGDI Signaling | 9.1E-04 | -1.897 | 0.09 | 1.3E-05 | -3.153 | 0.13 |
| Protein Ubiquitination Pathway | 8.3E-06 |  | 0.09 | 1.6E-05 |  | 0.11 |
| Paxillin Signaling | 2.1E-02 | 1.134 | 0.08 | 4.3E-05 | 1.604 | 0.15 |
| RAN Signaling | 4.9E-07 |  | 0.44 | 4.9E-05 |  | 0.38 |
| NRF2-mediated Oxidative Stress Response | 3.3E-03 | 1.414 | 0.08 | 6.2E-05 | 3.162 | 0.12 |
| Glycolysis I | 8.5E-03 |  | 0.17 | 7.1E-05 |  | 0.29 |
| Tight Junction Signaling | 6.3E-04 |  | 0.09 | 7.4E-05 |  | 0.12 |
| Gluconeogenesis I | 1.0E-02 |  | 0.16 | 9.5E-05 |  | 0.28 |
| DNA Double-Strand Break Repair by Non-Homologous End Joining | 1.1E-03 |  | 0.29 | 2.8E-04 |  | 0.36 |
| Pentose Phosphate Pathway (Oxidative Branch) | 6.8E-03 |  | 0.50 | 3.7E-04 |  | 0.14 |
| FAK Signaling | 2.9E-02 |  | 0.08 | 5.2E-04 |  | 0.14 |
| Phospholipase C Signaling | 1.5E-04 | 0.905 | 0.09 | 9.5E-04 | 0.832 | 0.10 |
| VEGF Signaling | 3.4E-02 | 1.342 | 0.08 | 7.4E-03 | 2.121 | 0.11 |
| Pentose Phosphate Pathway | 2.5E-04 |  | 0.40 | 8.9E-03 |  | 0.30 |
| Fcγ Receptor-mediated Phagocytosis | 4.8E-03 | 1.000 | 0.10 | 1.0E-02 | 3.162 | 0.11 |
| IL8 signalling | 1.1E-02 | 0.302 | 0.07 | 7.7E-02 | 2.530 | 0.07 |
| Acute phase response | 6.6E-02 | 1.890 | 0.06 | 2.2E-02 | 0.302 | 0.08 |
| Ketogenesis | 4.1E-03 |  | 0.30 |  |  |  |
| Fatty Acid β-oxidation I |  |  |  | 3.4E-05 |  | 0.28 |
| Oxidative Phosphorylation |  |  |  | 7.6E-05 |  | 0.14 |
| Cell Cycle: G2/M DNA Damage Checkpoint Regulation |  |  |  | 1.6E-03 | 2.236 | 0.16 |
| ERK/MAPK Signaling |  |  |  | 5.2E-03 | 0.832 | 0.09 |
| Glutathione redox reactions |  |  |  | 6.0E-03 |  | 0.67 |
| Rac Signaling |  |  |  | 7.4E-03 | 2.111 | 0.11 |
| PI3K/AKT Signaling |  |  |  | 9.5E-03 | 1.155 | 0.10 |
| Thioredoxin pathway |  |  |  | 1.9E-02 |  | 0.17 |
| fMLP signaling in neutrophils |  |  |  | 2.5E-02 | 2.121 | 0.09 |

**Table S6**

| **Toxicity Effects** | **Azathioprine 100 µM** | | **Taxol 100 nM** | |
| --- | --- | --- | --- | --- |
|  | **p value** | **Ratio** | **p value** | **Ratio** |
| Mitochondrial Dysfunction | 2.04E-04 | 0.10 | 7.24E-06 | 0.13 |
| NRF2-mediated Oxidative Stress Response | 7.24E-04 | 0.08 | 6.31E-05 | 0.11 |
| Cardiac Fibrosis | 3.55E-03 | 0.08 | 5.37E-04 | 0.11 |
| Cell Cycle: G2/M DNA Damage Checkpoint Regulation |  |  | 2.04E-03 | 0.16 |
| Hepatic Fibrosis | 5.89E-03 | 0.09 | 4.37E-03 | 0.12 |
| Increases Depolarization of Mitochondria and Mitochondrial Membrane |  |  | 5.13E-03 | 0.25 |
| Decreases Depolarization of Mitochondria and Mitochondrial Membrane | 6.17E-04 | 0.24 | 1.41E-02 | 0.19 |
| Fatty Acid Metabolism |  |  | 1.41E-02 | 0.10 |
| Swelling of Mitochondria |  |  | 2.40E-02 | 0.21 |
| Increases Transmembrane Potential of Mitochondria and Mitochondrial Membrane | 2.82E-02 | 0.10 | 2.57E-02 | 0.12 |
| Decreases Permeability Transition of Mitochondria and Mitochondrial Membrane | 2.24E-02 | 0.29 | 3.80E-02 | 0.29 |
| Oxidative Stress |  |  | 4.17E-02 | 0.11 |
| PPARα/RXRα Activation | 1.41E-02 | 0.07 | 4.68E-02 | 0.08 |
| Negative Acute Phase Response Proteins | 2.00E-03 | 0.38 | 4.90E-02 | 0.25 |
| Positive Acute Phase Response Proteins |  |  | 4.68E-02 | 0.13 |
| Decreases Transmembrane Potential of Mitochondria and Mitochondrial Membrane | 2.00E-02 | 0.08 |  |  |
| Hypoxia-Inducible Factor Signaling | 3.16E-02 | 0.09 |  |  |
